# Phenolic Compounds from *Hypericum cerastoides* (Spach) N. Robson: Dereplication via UHPLC-HRMS/MS, Isolation, Identification, and Preliminary Biological Evaluation Focusing on Radical-Scavenging, Anti-α-Glucosidase, and Pro-Lipase Activities

**DOI:** 10.3390/metabo15100643

**Published:** 2025-09-25

**Authors:** Zlatina Kokanova-Nedialkova, Yana Ilieva, Teodor Marinov, Paraskev T. Nedialkov

**Affiliations:** 1 Department of Pharmacognosy, Faculty of Pharmacy, Medical University of Sofia, 2 Dunav Str., 1000 Sofia, Bulgaria; zlatina.kokanova@pharmfac.mu-sofia.bg (Z.K.-N.); t.marinov@pharmfac.mu-sofia.bg (T.M.); 2 Department of Infectious Microbiology, The Stephan Angeloff Institute of Microbiology, Bulgarian Academy of Sciences, 26 Acad. G. Bonchev Str., 1113 Sofia, Bulgaria; illievayana@gmail.com

**Keywords:** *Hypericum cerastoides*, LC-MS/MS, phenolic compounds, anti-α-glucosidase activity, pro-lipase activity, DPPH, ABTS

## Abstract

**Background/Objectives:** *Hypericum cerastoides* (Spach) N. Robson is a lesser-known species with potential pharmacological importance. This study aimed to profile phenolic compounds in its aerial parts and assess biological activities of isolated constituents, focusing on radical-scavenging, anti-α-glucosidase, and pro-lipase effects. **Methods:** Phenolic compounds from *H. cerastoides* aerial parts were dereplicated via UHPLC-HRMS/MS. The structures of isolated compounds were determined using spectroscopic methods (1D and 2D NMR, UV, and HRMS-ESI). Radical-scavenging was evaluated by DPPH and ABTS assays; anti-α-glucosidase and pro-lipase activities were measured by LC-MS. **Results:** UHPLC-HRMS profiling of a hydroalcoholic extract tentatively identified and quantified 39 phenolic compounds, mainly flavonoids and hydroxycinnamic acid derivatives. Furthermore, two new phenolic compounds, namely hypercerastoside A (**HC4**) and hypercerastoside B (**HC6**), together with three known compounds, coumaroylquinic acid (**HC1**), myricetin-3-O-glycoside (**HC2**), and myricetin-3-O-galactoside (**HC3**), as well as two artifacts, namely methyl ester of chlorogenic acid (**HC5**) and hypercerastoside C (**HC7**), were isolated from the ethylacetate extract of the aerial parts of title plant. Compounds **HC2**, **HC3**, and **HC5** displayed the highest radical-scavenging activity. The anti-α-glucosidase test showed that compounds **HC1** (IC_50_ = 44 µM) and **HC3** (IC_50_ = 206 µM) possessed similar activity to acarbose (IC_50_ = 206 µM). Myricetin glycosides **HC2** and **HC3** enhanced lipase activity fivefold at 200 µM. **Conclusions:** *H. cerastoides* is a promising source of bioactive phenolic compounds with significant radical-scavenging and enzyme-modulating activities. These preliminary findings support further exploration of its therapeutic potential, especially for oxidative stress-related disorders, type 2 diabetes, and cachexia.

## 1. Introduction

The genus *Hypericum* L. (St. John’s wort, Hypericaceae) comprises nearly 500 species that occur naturally or have been introduced on every continent of the world, except Antarctica [1]. Many of its representatives have been traditionally used as medicinal plants in different parts of the world, providing bioactive compounds with antidepressant, cytotoxic, antimicrobial, and antioxidant properties. Several groups of phenolic compounds have been identified in *Hypericum* species, including anthraquinone derivatives (naphthodianthrones and dianthrones), acylphloroglucinols, xanthones, benzophenones, flavonoids, and chromones [2,3]. *Hypericum cerastoides* (Spach) N. Robson (sect. Campylopus) is a perennial herbaceous plant, distributed in Southern Bulgaria, Northeastern Greece, and Northwestern Türkiye [4]. In Turkish folk medicine, the plant’s aerial parts are used for treating gastric disorders as a decoction or by drinking crushed juice every morning for 3 days [5,6]. A GC-MS analysis of the aerial parts of *Hypericum cerastoides* resulted in the characterization of 61 volatile constituents with α-pinene (58%), undecane (5%), and β-pinene (3%) as the most prominent compounds [7]. The essential oil (EO) obtained from the aerial parts of *Hypericum cerastoides* was recently analyzed using GC–MS–FID. Its composition was found to be notably distinct from that of other *Hypericum* species, including *H. perforatum*, *H. rumeliacum*, *H. montbretii*, and *H. calycinum*. The major constituents of the hydro-distilled EO from *H. cerastoides* were ethyl 2-methylpentanoate (6.87%), thymol (8.31%), hexadecanoic acid (36.48%), and 3,7,11,15-tetramethyl-2-hexadecen-1-ol (28.49%). Due to the limited available data on the EO composition of *H. cerastoides*, this study represents the first detailed report in the literature [8]. Isomangiferin was reported for *H. cerastoides* by Kitanov and Nedialkov in 1998 [9], while rutin was later detected in the same species by HPLC analysis conducted by Crockett et al. [10]. Further studies of the title plant revealed the presence of hypericin, pseudohypericin, garcimangosone D, 1,7-dihydroxy-5,6-dimethoxyxanthone, I3-II8-biapigenin, isoquercitrin, quercetin, (−)-epicatechin, 6-O-p-coumaroyl-α/β-glucopyranose, chlorogenic acid, daucosterol linoleate, cerastioside A (a normonoterpene glycoside), and cerastioside B (a benzophenone glycoside). The results also showed that cerastioside A exhibited weak cytotoxicity against A375 (skin) and MCF7 (breast) cell lines with IC_50_ values of 158.92 ± 16.74 and 198.41 ± 6.48 μM, respectively [11]. The present study focuses on dereplicating polar phenolic constituents in aerial parts of the title plant by UHPLC-HRMS/MS analysis, isolation, and structural elucidation of two new, three known, and two artifact phenolic compounds. The isolated compounds’ anti-α-glucosidase, pro-lipase, and radical-scavenging (DPPH and ABTS tests) activities were established.

## 2. Materials and Methods

### 2.1. General Experimental Procedures

Optical rotations were measured on a Rudolph Research Analytical Autopol VI (Hackettstown, NJ, USA). UV spectra were obtained using a UV-VIS spectrometer, Biochrom Libra S70 (Cambourne, UK). ESI-HRMS spectra were recorded using a Thermo Scientific Q Exactive Plus mass spectrometer (Thermo Fisher Scientific, Bremen, Germany). ^1^H- and ^13^C-NMR spectra were recorded on a Bruker Avance II+ 600 spectrometer at 300 K, in acetone-d_6_ (99.96%, Deutero GmbH, Kastellaun, Germany). Column chromatography (CC) was performed using MCI GEL CHP20P Polymeric Adsorbent (Supelco, Bellefonte, PA, USA) and Sephadex LH-20 (Supelco). Semi-preparative HPLC was performed on a Waters (Milford, MA, USA) Breeze 2 high-pressure binary gradient system, consisting of a pump model 1525EF, a manual injector model 7725i, and a UV detector model 2489. Separations were achieved on a semi-preparative HPLC column, Kromasil C18 (250 × 21.2 mm, 10 µm), at 18 mL/min, and on a Kromasil C18 (250 × 10 mm, 5 µm) column at 5 mL/min, both purchased from Eka Chemicals AB (Bohus, Sweden).

### 2.2. Plant Material

The aerial parts (flowers, leaves, and stems) of *Hypericum cerastoides* (Spach) N. Robson were collected near hut Treshtenik (GPS coordinates: 42.080921, 23.619284), Rila Mountain, Bulgaria, in July 2018 and were identified by P. T. Nedialkov. The voucher specimens (SOM-Co-1343) were deposited at the National Herbarium of the Institute of Biodiversity and Ecosystem Research (IBER) at the Bulgarian Academy of Sciences (BAS).

### 2.3. Extraction and Isolation

The aerial parts of *H. cerastoides* were dried in the shade for 2 weeks, and a powdered plant material (378 g, 9% on drying) was extracted at room temperature with CH_2_Cl_2_ (6 × 1.5 L) by percolation. The CH_2_Cl_2_ extracts were combined, and the solvent was evaporated under reduced pressure to yield 154 g of a dark-green, waxy residue. The resulting residue was suspended in 600 mL of water and then extracted with ethyl acetate (10 × 250 mL). The ethyl acetate layers were combined and evaporated to dryness in a vacuum. The resulting EtOAc residue (14.8 g) was divided into three equal portions, which were subjected to MCI gel (330 × 40 mm, 100 mL) eluted with a mobile phase of MeOH: water (10:90→60:40). Sixty fractions from each portion were combined into six pooled fractions (I–VI) based on LC-MS profiles. Fraction III (233 mg, 20–25% MeOH) was subjected to column chromatography (CC) over Sephadex LH-20 (800 × 30 mm, 25 mL) using methanol as the eluent. The compound **HC1** (3.78 mg) crystallized from sub-fraction 4–5 (45 mg). Semi-prep. HPLC of sub-fraction 14–18 (142 mg) eluted isocratically with MeCN-0.05% H_3_PO_4_ (12:88) led to the isolation of **HC2** (19 mg) and **HC3** (8 mg). Fraction IV (1279 mg, 25–35% MeOH) was subjected to column chromatography (CC) over Sephadex LH-20 (800 × 30 mm, 25 mL) using methanol as the eluent. An isocratic semi-prep. HPLC purification of subfraction 5–7 (682 mg) with MeCN-0.05% H_3_PO_4_ (20:80) gave pure **HC4** (361 mg) and **HC5** (33 mg). Furthermore, the fraction VI (671 mg, 45–50% MeOH) was subjected to CC over Sephadex LH-20 (800 × 30 mm, 25 mL) with MeOH as the eluent. Semi-prep. HPLC of sub-fraction 31–36 (119 mg) eluted isocratically with MeCN-0.05% H_3_PO_4_ (25:75) led to the isolation of **HC6** (16 mg) and **HC7** (17 mg).

#### 2.3.1. Hypercerastoside A (**HC4**) (4,6-Dihydroxy Benzophenone-2-*O*-β-D-2″-Acetylglucopyranoside)

White amorphous powder; ${\left[\alpha \right]}_{D}^{20}$: −45.6° (c 0.1, MeOH); UV/VIS *λ*_max_ (MeOH) nm (log ε): 207 (4.67), 251 (4.05), 298 (3.50); +AlCl_3_: 278, 338; +NaOAc: 348; ESI-HRMS: found *m*/*z* 433.1139 [M–H]^−^, calcd. for C_21_H_21_O_10_ *m*/*z* 433.1129; MS^2^: *m*/*z* 391.1054 [M–C_2_H_2_O]^−^, 373.0929 [M–AcOH–H]^−^, 229.0503 [M_agl_–H]^−^; ^1^H NMR (acetone-*d*_6_, 600 MHz): (Table 1); ^13^C NMR (acetone-*d*_6_, 150 MHz): (Table 1).

#### 2.3.2. Hypercerastoside B (**HC6**) (4-*O*-{6-[(2*E*)-*p*-Hydroxycinnamoyl]-β-D-Glucopyranosyl}-6-isopropyl-tetrahydro-2*H*-pyran-2-one)

White amorphous powder; ${\left[\alpha \right]}_{D}^{20}$: +1.66° (c 0.115, CH_3_CN); UV/Vis λ_max_ (CH_3_CN) nm (log ε): 226 (4.17), 311 (4.38); ESI-HRMS: found *m*/*z* 467.1909 [M + H]^+^, calcd. for C_23_H_31_O_10_ *m*/*z* 467.1912, MS^2^: *m*/*z* 309.0963 [M–M_agl_–H_2_O + H]^+^, 303.1433 [M–C_9_H_7_O_3_]^+^, 159.1016 [M_agl_ + H]^+^, 147.0440 [M_pCA_−OH]^+ 1^H NMR (acetone-d_6_, 600 MHz): (Table 2); ^13^C NMR (acetone-d_6_, 150 MHz): (Table 2).

#### 2.3.3. Hypercerastoside C (**HC7**) (Methyl 3-*O*-{6-[(2*E*)-*p*-hydroxycinnamoyl]-β-glucopyranosyl}-6-methyl-5-hydroxyheptanoate)

White amorphous powder; ${\left[\alpha \right]}_{D}^{20}$: +0.92 (c 0.110, CH_3_CN); UV/Vis λ_max_ (CH_3_CN) nm (log ε): 227sh (4.29), 313 (4.40); ESI-HRMS: found *m*/*z* 499.217 [M + H]^+^, calcd. for C_24_H_35_O_11_ *m*/*z* 499.2174, MS^2^: *m*/*z* 309.0966 [M–M_agl_–H_2_O + H]^+^, 191.1278 [M_agl_ + H_2_O + H]^+^, 173.1172 [M_agl_−OH]^+^, 147.0440 [M_pCA_−OH]^+^; ^1^H NMR (acetone-d_6_, 600 MHz): (Table 3); ^13^C NMR (acetone-d*_6_*, 150 MHz): (Table 3).

### 2.4. Acid Hydrolysis

Compounds **HC4**, **HC6,** and **HC7** (each 4 mg) were separately refluxed with 2 mL of 2 N HCl-MeOH (1:1) for 2 h. The reaction mixtures were filtered through Amberlite IRC-86 resin and washed with H_2_O. The eluate was filtered through a Diaion HP-20SS column and eluted with H_2_O and MeOH. The water portions were evaporated to dryness and analyzed for sugars by HPLC, following the method of Tanaka et al., with some modifications [12,13]. Briefly, the dry water eluate was treated with a solution (0.1 mL) of L-cysteine methyl ester in pyridine (5 mg/mL) at 60 °C for one hour. A solution (0.1 mL) of o-tolylisothiocyanate in pyridine (5 mg/mL) was added to the mixture and heated at 60 °C for 1 h. The solution was analyzed using HPLC [Purospher STAR RP-18 5 μm column (Merck KGaA, Darmstadt, Germany; 4.6 × 250 mm) with 25% ACN in 50 mM H_3_PO_4_, flow rate 1 mL min^−1^, UV detection at 250 nm]. The presence of D-glucose (t_R_ values of the tolylthiocarbamoyl-thiazolidine derivatives was 18.7 min) was found in the residues.

### 2.5. Anti-α-Glucosidase Activity Assay

The *α*-Glucosidase inhibitory activity was determined by LC-MS using *p*-nitrophenyl-α-D-glucopyranoside as the substrate, as described by Kokanova-Nedialkova et al. [14]. The assay mixture (160 µL) was prepared accordingly to Kang et al. [15] with some modifications [14]. An aliquot of 20 µL of a sample in phosphate buffer (pH 6.8) containing 10% DMSO (or phosphate buffer containing 10% DMSO as a control), 100 µL phosphate buffer (pH 6.8), and 20 µL enzyme solution (0.2 U/mL α-glucosidase in phosphate buffer) were mixed and incubated at 37 °C for 15 min. Then, 20 µL of substrate solution (2.5 mM *p*-nitrophenyl-α-D-glucopyranoside, prepared in the same buffer) was added. The reaction was processed at 37 °C for 15 min and stopped by adding 840 µL acetonitrile (AcCN). The supernatant was then used for LC-MS analysis after centrifugation at 12,000 rpm for 10 min. The amount of p-nitrophenol released from *p*-nitrophenyl-α-D-glucopyranoside was quantified using liquid chromatography-tandem mass spectrometry (LC-MS). The inhibitory rates (%) were calculated according to the formula:

$$\%\;\alpha -Glucosidase\;inhibitory\;activity=\frac{A_{к}-A_{sample}}{A_{к}}\times 100,$$

where A_к_ is the area of the p-nitrophenol peak in the control, and A_sample_ is the area of the *p*-nitrophenol peak in the sample. Acarbose was used as a positive control for the α-glucosidase inhibition assay.

### 2.6. Assay for Modulation of Lipase Activity

This assay was adapted from previous reports [16] for the determination of anti-lipase and pro-lipase activity by LC-MS [14]. Briefly, lipase from porcine pancreas, Type II (Sigma-Aldrich, St. Louis, MA, USA, product L3126), was dissolved in ultra-pure water at a 5 mg/mL concentration. The supernatant was then used after centrifugation at 15,000 rpm for 10 min. The assay buffer was 100 mM Tris buffer (pH 8.2), and *p*-nitrophenyl laurate was used as the substrate. The substrate stock was 2 mg *p*-nitrophenyl laurate dissolved in 1 mL isopropanol and 4 mL 5 mM sodium acetate (pH 5.0) containing 1% Triton X-100 (Fisher Scientific, Bridgewater, NJ, USA). The mixture was heated to facilitate dissolution, thoroughly mixed, and then cooled to room temperature. The assay mixture (1 mL) contained 20 µL of a sample in DMSO (or DMSO as a control), 860 µL of Tris buffer (pH 8.2), and 30 µL of enzyme solution. The mixture was combined, shaken, and incubated at 37 °C for 15 min. Then, 90 µL of substrate solution was added and shaken again. The reaction was processed at 37 °C for 2 h and stopped by adding 800 µL CH_3_CN to 200 µL of the assay mixture. The supernatant was then used for LC-MS analysis after centrifugation at 15,000 rpm for 10 min. The amount of p-nitrophenol released from *p*-nitrophenyl laurate was quantified using LC-MS. Lipase inhibitory activity (%) was calculated according to the formula:

$$\%\;Lipase\;inhibitory\;activity=\frac{A_{к}-A_{sample}}{A_{к}}\times 100,$$

where A_к_ is the area of the p-nitrophenol peak in the control, and A_sample_ is the area of the *p*-nitrophenol peak in the sample. Orlistat was taken as a positive control for a lipase inhibition assay.

Pro-lipase activity (%) was calculated according to the formula:

$$\%\;Prolipase\;activity=\frac{A_{sample}-A_{k}}{A_{к}}\times 100,$$

where A_к_ is the area of the *p*-nitrophenol peak in the control, and A_sample_ is the area of the *p*-nitrophenol peak in the sample.

### 2.7. DPPH Radical-Scavenging Activity Assay

The scavenging activity of phenolic compounds against DPPH radicals was assessed using Kokanova-Nedialkova and Nedialkov methods, with some modifications [17]. Briefly, 300 µL of each compound in EtOH (100 µM) was mixed with 300 µL of DPPH ethanol solution (100 µM). The reaction mixture was thoroughly vortexed and left in the dark at room temperature for 30 min. The absorbance of the mixture was measured at 517 nm. The following equation calculates the ability to scavenge DPPH radicals:

$$\%DPPH\;radical\;scavenging\;activity=\frac{{\mathrm{Abs}}_{\mathrm{control}}-{\mathrm{Abs}}_{\mathrm{sample}}}{{\mathrm{Abs}}_{\mathrm{control}}}\times 100,$$

where Abs_control_ is the absorbance of the DPPH radical in EtOH, and Abs_sample_ is the absorbance of the DPPH radical solution mixed with a sample.

Positive controls were vitamin C (100 µM) and Trolox (100 µM).

### 2.8. ABTS Radical-Scavenging Activity Assay

For ABTS assay, the procedure followed the method of Kokanova-Nedialkova and Nedialkov with some modifications [17]. The stock solutions included 7 mM ABTS and 2.4 mM potassium persulfate. The working solution was then prepared by mixing the two stock solutions in equal quantities and allowing them to react for 12–14 h at room temperature in the dark. The solution was then diluted by mixing 1 mL of the ABTS solution with 30 mL of ethanol. A fresh ABTS solution was prepared for each assay. Furthermore, 300 µL of each compound in EtOH (100 µM) was allowed to react with 1 mL of the ABTS solution, and the absorbance was taken at 734 nm after 7 min using a spectrophotometer. The ABTS scavenging capacity of the compound was calculated as:

$$\%ABTS\;radical\;scavenging\;activity=\frac{{\mathrm{Abs}}_{\mathrm{control}}-{\mathrm{Abs}}_{\mathrm{sample}}}{{\mathrm{Abs}}_{\mathrm{control}}}\times 100$$

where Abs_control_ is the absorbance of the ABTS radical in ethanol, and Abs_sample_ is the absorbance of an ABTS radical solution mixed with a sample.

Vitamin C (100 µM) and Trolox (100 µM) were used as positive controls.

### 2.9. Statistical Analysis

The statistical program “Medcalc” ver. 23 (MedCalc Software Ltd., Ostend, Belgium) was used for data analysis. The results were expressed as mean ± standard deviation (SD) of three independent experiments, each performed in triplicate. The statistical significance of the results vs. respective controls was calculated employing a Mann–Whitney non-parametric test. The accepted statistically significant differences occurred when *p* < 0.05.

## 3. Results

### 3.1. Dereplication and Semi-Quantitative Determination of Polar Phenolic Compounds in Aerial Parts from Hypericum cerastoides by UHPLC-HRMS/MS Analysis

Dereplication and semi-quantitative determination of polar phenolic compounds from the aerial parts of *Hypericum cerastoides* were achieved utilizing UHPLC-HRMS/MS analysis [18]. Thirty-nine phenolic compounds were identified (Table S1): flavonol aglycones and their glycosides, flavan-3-ols and a flavolignan, hydroxycinnamic acid derivatives, benzophenones, xanthones, and flavone dimers.

#### 3.1.1. Flavan-3-ols and a Flavolignan

In the full MS spectrum, the deprotonated molecules [M−H]^−^ of **1** and **2** appeared at *m*/*z* 305.07. The MS^2^ spectra of both compounds showed similar product ions at *m*/*z* 179.03, 137.02, and 125.02 (Figure S22), resulting from pyrogallol neutral loss, retro-Diels-Alder (RDA) fragmentation, and heterocyclic ring fusion (HFR). However, there was a substantial difference in retention times of **1** and **2,** which were 2.87 and 4.98 min., respectively. Thus, comparing the obtained MS^2^ spectra and the retention times with recent data reports, metabolites **1** and **2** were tentatively identified as gallocatechin and galloepicatechin [19]. The deprotonated molecule [M−H]^−^ of compound **31** appeared at *m*/*z* 451.10. Its MS^2^ spectrum showed characteristic product ions at *m*/*z* 341.07, 217.01, 189.02, 109.03, 231.03 (Figure S23). The loss of the catechol group (110 Da) attached to the pyranone ring led to the formation of a fragment at *m*/*z* 341.07, which further lost a second catechol moiety (ring B from the catechin skeleton) and produced the ion at *m*/*z* 231.03. Subsequently, fragmentation of the later ion showed CH_3_ loss, followed by CHO elimination to give the product ions at *m*/*z* 217.01 and 189.02, respectively, which was in conformance with the fragmentation pattern of the cinchonain I isomers [20,21]. Thus, metabolite **31** was tentatively identified as cinchonaine Ib.

#### 3.1.2. Hydroxycinnamic Acid Derivatives

The deprotonated molecule [M−H]^−^ of compound **5** appeared at *m*/*z* 325.09 in the full MS scans. The MS^2^ spectrum showed the product ions at *m*/*z* 163.04 and 179.17 corresponding to a loss of the hexose (162 Da) and a deprotonated form of a hexose unit, respectively. Subsequently, the former ion produced a fragment at *m*/*z* 119.05, indicative of carboxyl (44 Da) loss, characteristic of the cinnamic acids (Figure S24). Thus, according to the literature data, metabolite **5** was tentatively identified as 4-*O*-β-D-glucosyl-4-coumaric acid [22].

#### 3.1.3. Flavonol Aglycones and Their Glycosides

The deprotonated molecule [M−H]^−^ of compound **37** appeared at *m*/*z* 285.04 in the full MS scans. Its MS^2^ spectrum showed characteristic product ions at *m*/*z* 257.05, 239.03, 229.05, and 185.06 (Figure S25). The former product ion resulted from CO (28 Da) loss. Subsequently, it undergoes further fragmentation, resulting in fragment ions at *m*/*z* 239.03 and 229.05, corresponding to the loss of H_2_O (18 Da) and CO (28 Da), respectively. Furthermore, the later fragment ion loses a CO_2_ (44 Da), producing a fragment at *m*/*z* 185.06. According to the literature data [23], the metabolite **37** was identified as kaempferol. Compound **26** shows a deprotonated [M−H]^−^ molecule at *m*/*z* 593.15. Its MS^2^ spectrum showed fragment ions at *m*/*z* 416.60, 285.04, 284.03, 257.05, 239.05, and 229.05. The former ion resulted from splitting off an *O*-linked hexose from a rhamnose unit. Subsequently, the loss of rhamnosyl moiety gave the base peak (*m*/*z* 285.04) corresponding to the deprotonated form of an aglycone. In contrast, a fragment ion at *m*/*z* 284.03 was derived from homolytic cleavage of the glycosidic bond [24]. In addition, the product ions at *m*/*z* 257.05, 239.05, and 229.05 were typical for kaempferol. Thus, metabolite **26** was tentatively identified as kaempferol-O-hexosylrhamnoside. The deprotonated molecule [M−H]^−^ of compound **18** appeared at *m*/*z* 609.15. Its MS^2^ spectrum showed a fragment ion at *m*/*z* 301.04, corresponding to the deprotonated molecule of the aglycon, and a fragment ion at *m*/*z* 300.03 (the base peak) derived from [M−H−Rha−Glu−H]^−^, the homolytic cleavage of the glycosidic bond. Furthermore, the latter ion undergoes subsequent losses of CHO (29 Da), CO + OH (45 Da), and CHO + CO (57 Da) to give the product ions at *m*/*z* 271.03, 255.03, and 243.03, respectively. According to the literature data [25], the metabolite was tentatively identified as quercetin-3-*O*-rutinoside (rutin). The deprotonated molecules [M–H]^−^ of the isobaric compounds **23** and **27** appeared at *m*/*z* 505.10. Their MS^2^ spectra showed similar fragment ions at *m*/*z* 301.04, 300.03, 271.03, 255.03, and 243.03, which are typical for quercetin. Furthermore, the product ions [M–H–CH_3_CO]^−^ at *m*/*z* 463.09 for **23** and [M–H–CH_3_COO]^−^ at *m*/*z* 445.08 for **27** indicated an acetyl group content in the hexose moiety. Thus, metabolites **23** and **27** were tentatively identified as quercetin-3-O-(6″-O-acetyl)-galactoside and quercetin-3-O-(2″-*O*-acetyl)-glucoside [26,27], respectively. Compounds **29** and **34** are also isobaric and show deprotonated [M–H]^−^ molecules at *m*/*z* 463.09. The MS^2^ spectra showed fragment ions at *m*/*z* 301.04 and 300.03, corresponding to the neutral loss of the hexose as a sugar moiety, characteristic of *O*-glycosides. The former ion corresponded to the deprotonated molecule of the aglycone, while the latter resulted from homolytic cleavage of the glycosidic bond. In addition, both compounds formed a fragment ion at *m*/*z* 151.00 with high intensity due to ^1,3^A_0_ cleavage. According to literature data [28], the greater abundance of the ion at *m*/*z* 301.04 compared to that at *m*/*z* 300.03, a prominent ion at *m*/*z* 151.00 (Figure S26), and the absence of more pronounced losses of small molecules such as OH, CO, HCO, etc., is observed in 4′-*O*-glycosides. Thus, compounds **29** and **34** were tentatively identified as quercetin-4′-*O*-galactoside and quercetin-4′-*O*-glucoside. The deprotonated molecule [M−H]^−^ of compound **13** appeared at *m*/*z* 625.14 in the full MS scans. The MS^2^ spectrum showed a fragment ion at *m*/*z* 317.03 resulting from a neutral loss of 309 Da, indicative of an O-linked rutinose. Furthermore, the compound produced a fragment ion at *m*/*z* 316.03 (the base peak) resulting from the homolytic cleavage of the glycosidic bond, which subsequently underwent CO + OH (45 Da) loss to give an ion at *m*/*z* 178.99, corresponding to ^1,2^A_0_ cleavage. According to a recent data report, metabolite **13** was tentatively identified as myricetin-*O*-rutinoside [29]. Compound **16** shows a deprotonated [M−H]^−^ molecule at *m*/*z* 565.08, which undergoes subsequent loss of CO_2_ (44 Da) to give an ion at *m*/*z* 521.09 in full MS scans. This phenomenon is typical for the malonyl glycosides, which are very labile in the negative ion mode using electrospray ionization [30]. In the MS^2^ spectrum, the later ion loses acetylhexose (205 Da) to produce an ion at *m*/*z* 316.02 (base peak). Furthermore, the fragment ions at *m*/*z* 317.03 and 178.99 are characteristic of myricetin. Thus, metabolite **13** was tentatively identified as myricetin-O-(malonyl)-hexoside. The deprotonated [M−H]^−^ molecules of isobaric compounds **20**, **21**, and **32** appeared at *m*/*z* 479.08. Their MS^2^ spectra show similar fragment ions at *m*/*z* 317.04, 316.04, 178.99, and 151.00. The precursor ions showed neutral losses of 163 Da to give a fragment ion at *m*/*z* 317.04 (the base peak) corresponding to a deprotonated myricetin molecule. In contrast, the ion at *m*/*z* 316.04 resulted from homolytic cleavage of the glycosidic bond. The former ion subsequently undergoes ^1,2^A_0_ and ^1,3^A_0_ cleavages in ring C to give fragment ions at *m*/*z* 178.99 and 151.00, respectively. According to the literature data [31], the higher abundance of the ion at *m*/*z* 317.04 compared to that at *m*/*z* 316.04 indicated that the hexose unit is linked to 7-OH. Furthermore, the difference in retention times of compounds **20**, **21**, and **32**, which were 14.30, 14.50, and 10.94 min, respectively, suggested the presence of a different type of hexose moiety. Thus, metabolites **20**, **21**, and **32** were tentatively identified as myricetin-7-O-galactoside, myricetin-7-*O*-glucoside, and myricetin-7-*O*-hexoside.

#### 3.1.4. Benzophenones

The deprotonated molecule [M−H]^−^ of compound **14** appeared at *m*/*z* 391.10 in the full MS scans. The MS^2^ spectrum showed a fragment ion at *m*/*z* 229.05, resulting from a neutral loss of 162 Da, indicative of an *O*-linked hexose. Furthermore, the latter ion undergoes cleavage of the bond between the carbonyl function and the aromatic ring B to give the fragment ion at *m*/*z* 151.00 (Figure S27). Thus, metabolite **14** was tentatively identified as garcimangosone D [32]. Compound **35** showed a deprotonated molecule [M−H]^−^ at *m*/*z* 433.11. Its MS^2^ spectrum produced a fragment ion at *m*/*z* 391.10 due to the loss of a CH_3_CO group, indicating the presence of an acetyl group in a hexose moiety. Furthermore, the fragmentation pattern was identical to that of compound **14**. Thus, metabolite **37** was tentatively identified as trihydroxybenzophenone-*O*-(acetyl)-glucoside (acetyl-garcimangosone D).

In addition, Table S1 shows the results of the LC-HRMS analysis of metabolites **2–4**, **6–12**, **15**, **17**, **19**, **22**, **24**, **28**, **30**, **33**, **35**, **36**, **38**, and **39**. A detailed description of their fragmentation patterns is given here [18].

### 3.2. Isolation and Identification of Some Polar Phenolic Compounds from the Ethylacetate Extract of the Aerial Parts of Hypericum cerastoides

**Figure 1:**
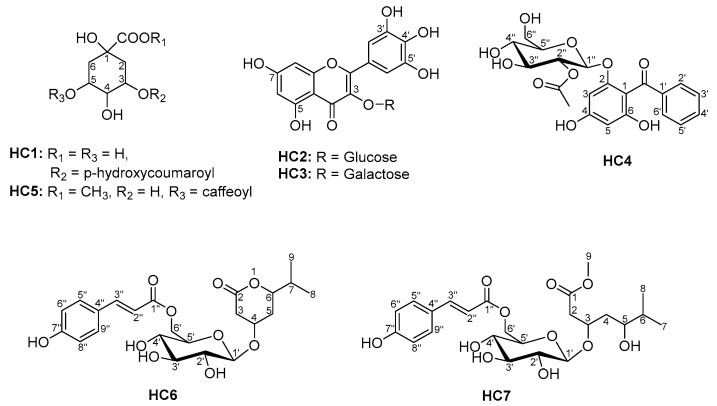
The structures of the isolated phenolic compounds from the ethyl acetate extract of *H. cerastioides*.

An extensive chromatographic procedure of the EtOAc extract from the aerial parts of *Hypericum cerastoides* (Spach) N. Robson resulted in the isolation and structural identification of seven phenolic compounds (**HC1**–**HC7**) (Figure 1). The compounds’ structures were elucidated using spectral (MS, UV, and NMR) methods and hydrolysis. The ^1^H and ^13^C NMR spectra signals were unambiguously assigned using 2D NMR techniques. The known compounds were identified as coumaroylquinic acid **HC1**, myricetin-3-O-glycoside **HC2**, myricetin-3-O-galactoside **HC3**, and methyl ester of chlorogenic acid **HC5**, respectively, by comparing their spectroscopic data with those reported in the literature [33,34,35,36].

**Figure 2:**
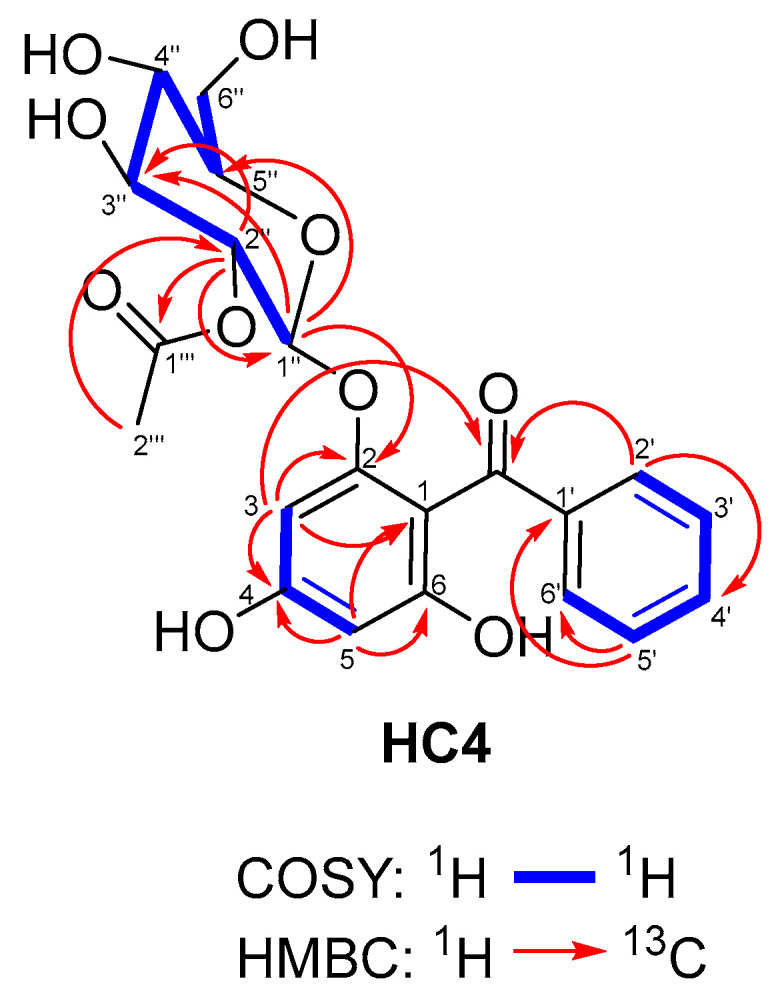
The selected COSY and HMBC correlations of **HC4**.

Compound **HC4** (The isolation and structural elucidation of compound **HC4** were part of Y.I.’s PhD Thesis, which can be found at https://ras.nacid.bg/api/reg/FilesStorage?key=cffffa1b-ac29-4f17-a9a7-1a998722ba7b&mimeType=application/pdf&fileName=Disertacia-Yana_Ilieva.pdf&dbId=1 (in Bulgarian), accessed on 15 August 2025 [37]) was obtained as an optically active, colorless amorphous powder. The ESI-HRMS spectrum of **HC4** in negative mode showed a deprotonated molecule [M–H]^−^ at *m*/*z* 433.1139, suggesting a molecular formula C_21_H_22_O_10_. The MS/MS experiment revealed sequential neutral loss of an acetic acid (433.11→373.09) and a hexose (373.09→229.05). The ^1^H NMR spectrum (Table 1) displayed five signals corresponding to aromatic protons. The three of these that appeared downfield as multiplets at *δ*_H_ 7.72 (2H, H-2′ and H-6′), 7.55 (1H, H-4′), and 7.44 (2H, H-3′ and H-5′) shared a single COSY network (Figure 2). These signals exhibited positive cross-peaks in the HSQC experiment, corresponding to the carbon signals in the ^13^C NMR spectrum (Table 1), which appeared at *δ*_C_ 129.9 (C-2′ and C-6′), 133.1 (C-4′), and 128.9 (C-3′ and C-5′). These signals, together with the signal of non-oxygenated quaternary carbon at *δ*_C_ 140.3 (C-1′), were attributed to an unsubstituted phenyl group. The C-1′ carbon was connected to the carbonyl function at *δ*_C_ 196.5 (C=O) since some protons of the unsubstituted phenyl ring correlated with its signal in the HMBC experiment (Figure 2). In addition, the ^1^H NMR spectrum showed another two aromatic proton signals that appeared upfield as *m*-coupled doublets at *δ*_H_ 6.32 (1H, H-3) and 6.18 (1H, H-5), which correlated in the HSQC experiment with the signals at *δ*_C_ 95.8 (C-3) and 98.0 (C-5), respectively. In addition to the latter signals, the ^13^C NMR spectrum also shows signals of four quaternary carbons at *δ*_C_ 162.4 (C-4), 160.0 (C-6), 158.6 (C-2), and 109.4 (C-1), of which the first three are oxidized, while the last one is linked to the carbonyl function at *δ*_C_ 196.5. The signals discussed so far were typical of benzophenones with a 2,4,6-oxygenation pattern. In addition to the benzophenone signals, the ^1^H spectrum showed peaks at *δ*_H_ 5.01 (1H, d, *J* = 8.0 Hz, H-1″), 4.46 (1H, dd, *J* = 8.0, 9.6 Hz, H-2″), 3.55 (1H, dd, *J* = 9.6, 9.0 Hz, H-3″), 3.50 (1H, m, H-5″), 3.41 (1H, dd, *J* = 8.9, 9.6 Hz, H-4″), 3.90 (1H, dd, *J* = 2.6, 11.9 Hz, Ha-6″) and 3.71 (1H, dd, *J* = 5.6, 11.9 Hz, Hb-6″) that showed cross-peaks in HSQC experiment with the signals at *δ*_C_ 99.6 (C-1″), 73.7 (C-2″), 75.9 (C-3″), 77.9 (C-5″), 71.3 (C-4″), 62.4 (C-6″), respectively, belonging to glucopyranose unit in β-configuration.

According to the correlation of the signal of the anomeric proton (H-1″) with the signal at *δ*_C_ 158.6 (C-2) in the HMBC experiment, C-2 was identified as the position of the sugar moiety. Furthermore, the ^1^H NMR spectrum showed a triproton singlet at *δ*_H_ 1.78 assigned to the methyl group of the acetyl moiety that was represented in the ^13^C-NMR spectrum by the signals at *δ*_C_ 169.3 (C-1′′′′) and 20.7 (C-2′′′′). These signals formed a cross-peak network in the HMBC experiment with the signals of H-2″ and C-2″, thus pointing out that the second OH-group of the glucopyranose unit was esterified with acetic acid. Therefore, the structure of **HC4** was established as 4,6-dihydroxybenzophenone-2-*O*-β-D-2ꞌꞌ-acetylglucopyranoside and was given the trivial name hypercerastoside A.

**Figure 3:**
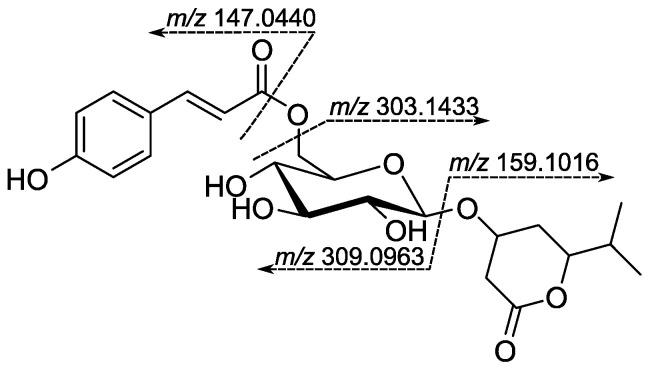
A plausible fragmentation of compound **HC6**.

Compound **HC6** was obtained as an optically active, colorless amorphous powder. The ESI-HRMS spectrum of **HC6** in positive mode showed a protonated molecule [M + H]^+^ at *m*/*z* 467.1909 (calculated 467.1912 for C_23_H_29_O_10_), suggesting a molecular formula C_23_H_30_O_10_ with nine degrees of unsaturation. The protonated molecule’s MS/MS spectrum showed characteristic fragments at *m*/*z* 309.0963 and 303.1433 due to aglycone and acyl moiety neutral loss, respectively. Furthermore, the product ions at *m*/*z* 159.1016 and 147.0440 can be attributed to the protonated molecule of the aglycone and *p*-hydroxycoumaroyl fragment, respectively. The plausible fragmentation is given in Figure 3.

**Figure 4:**
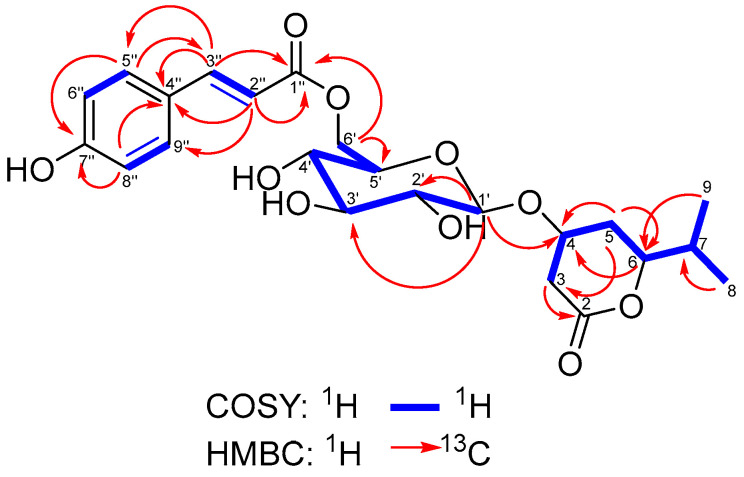
Selected COSY and HMBC correlations of **HC6**.

The ^1^H-NMR spectrum of **HC6** (Table 2) showed signals of two vicinal methyl groups (at *δ*_H_ 0.91) that gave a correlation in the COSY experiment (Figure 4) with a methine at *δ*_H_ 1.80. The latter was adjacent to an oxymethine at *δ*_H_ 4.45, which is a part of a hydrocarbon chain consisting, in addition, of two methylene groups (*δ*_H_ 2.27, 1.74, and 2.67), divided by an oxymethine at *δ*_H_ 4.35. Furthermore, in the ^13^C NMR spectrum (Table 2), along with the hydrogen-bearing carbons involved in the hydrocarbon chain (*δ*_C_ 17.7, 18.4, 31.7, 33.1, 37.0, 72.9, and 80.9), there was a carbonyl (*δ*_C_ 169.8) associated with an ester linkage.

These ^1^H and ^13^C NMR data agreed with those for 4-hydroxy-6-isopropyltetrahydro-2*H*-pyran-2-one, the aglycone of cerastioside A [11]. Some sugar signals also appeared in the ^1^H NMR spectrum of **HC6**: the double doublets at *δ*_H_ 3.24, 3.45, and 3.40 with axial coupling constants, a multiplet at *δ*_H_ 3.61, an oxymethylene at *δ*_H_ 4.52 and 4.29, and a signal for an anomeric proton at *δ*_H_ 4.50 in *β*-configuration, all typical for a glucopyranose unit. The latter signal gave an HMBC correlation (Figure 4) with C-4 at *δ*_C_ 72.9, which was evidence for the attachment position of the glucopyranosyl unit to the aglycone. In addition, the ^1^H NMR spectrum showed two doublets, each representing two protons at *δ*_H_ 6.91 and 7.57, which were typical for a para-substituted benzene ring. Furthermore, the olefinic proton signals observed at *δ*_H_ 6.39 and 7.64 with a coupling constant of *J* = 16 Hz indicated a double bond with *trans*-configuration. Both signals gave HMBC correlations with benzene ring carbons at *δ*_C_ 126.9 and 131.0 and with the carbonyl at *δ*_C_ 167.5. These NMR data completely agreed with an esterified trans p-coumaric acid [38]. The acid carbonyl signal (*δ*_C_ 167.5) gave HMBC correlations with the sugar protons at position 6′ (*δ*_H_ 4.52 and 4.29). Thus, the structure of **HC6** was established as 4-*O*-{6-[(2*E*)-*p*-hydroxycinnamoyl]-β-D-glucopyranosyl}-6-isopropyl-tetrahydro-2*H*-pyran-2-one and was given the trivial name hypercerastoside B.

**Figure 5:**
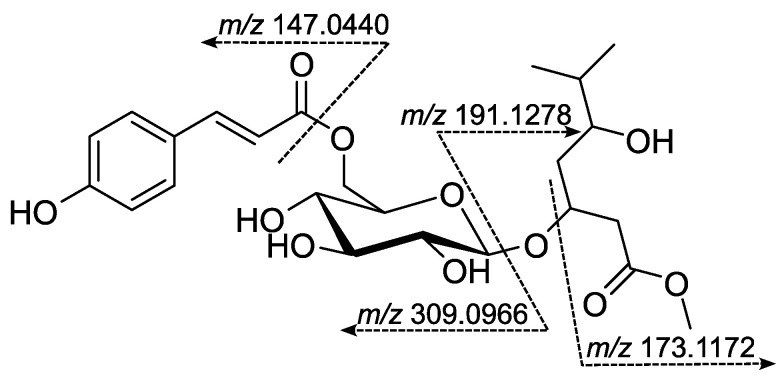
A plausible fragmentation of compound **HC7**.

Compound **HC7** was obtained as an optically active, colorless amorphous powder. The ESI-HRMS spectrum of **HC7** in positive mode showed a protonated molecule [M + H]^+^ at *m*/*z* 499.2170 (calculated 499.2174 for C_24_H_35_O_11_), suggesting a molecular formula C_23_H_30_O_10_ with eight degrees of unsaturation. The MS/MS spectrum of the protonated molecule of **HC7** shared two fragments (*m*/*z* 147.0440 and 309.0966) with those of **HC6**. This was evidence that both compounds had common moieties in their molecules. Furthermore, the characteristic product ions at *m*/*z* 191.1278 and 173.1172 were due to the aglycone moiety. The plausible fragmentation pattern of **HC7** is shown in Figure 5.

**Figure 6:**
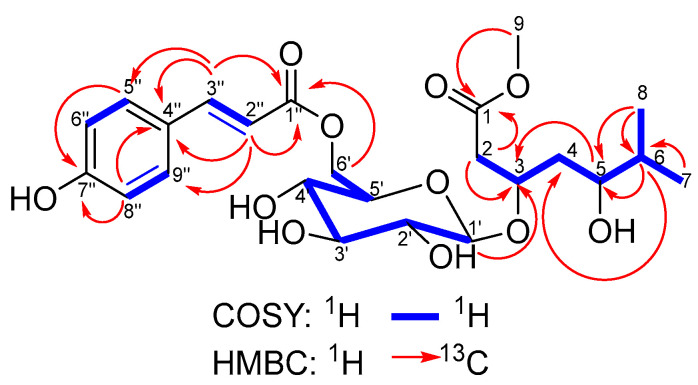
Selected COSY and HMBC correlations of **HC7**.

The ^1^H NMR spectrum of compound **HC7** (Table 3) displays signals corresponding to the sugar moiety (*δ*_H_ 4.49, 3.18, 3.45, 3.37, 3.61, 4.52, and 4.27) and *p*-hydroxycinnamic acid (*δ*_H_ 6.40, 7.63, 7.57, and 6.91), which are comparable to those observed in compound **HC6**. A similar correspondence is noted in the ^13^C NMR spectra of both compounds. Furthermore, analogous COSY and HMBC correlations are observed (Figure 6), supporting a glucopyranose unit esterified at the C-6 position with *p*-hydroxycinnamic acid, as in **HC6**. The ^1^H NMR spectrum of **HC7** also indicates the presence of a terminal isopropyl group, as evidenced by two geminal methyl signals, H_3_-7 (*δ*_H_ 0.85) and H_3_-8 (*δ*_H_ 0.83), and a methine signal H-6 (*δ*_H_ 1.64). These signals are shifted upfield by 0.06, 0.08, and 0.16 ppm, respectively, compared to **the HC6** signal. The H-6 signal exhibits a COSY correlation with the oxymethine proton H-5 (*δ*_H_ 3.54), which is shifted upfield by 0.91 ppm. The adjacent methylene protons H_2_-4 (*δ*_H_ 1.78 and 1.69) are also upfield-shifted, by 0.49 and 0.05 ppm, respectively. The remaining proton signals in the spectrum of **HC7**, including the oxymethine proton H-3 (*δ*_H_ 4.30) and the methylene protons H_2_-2 (*δ*_H_ 2.65), differ only slightly from those in **HC6**. In the ^13^C NMR spectrum of **HC7**, the aglycone signals exhibit upfield and downfield shifts relative to those of **HC6**. The most pronounced downfield shift (Δ*δ* = +7.1 ppm) is observed for the C-5 carbon adjacent to the oxymethine group, while the most significant upfield shift (Δ*δ* = −8.6 ppm) occurs for the C-6 methylene carbon. These observations suggest that in **HC7**, the hydroxyl group at C-5 is free and not involved in an ester linkage.

In addition, a singlet integrating for three protons is observed at *δ*_H_ 3.64, which shows an HMBC correlation with the carbonyl carbon at C-1 (δ_C_ 172.8). The HMBC spectrum also reveals a correlation between the anomeric proton of the sugar unit (*δ*_H_ 4.49) and the methine carbon at C-3 (*δ*_C_ 76.8), confirming that the sugar is attached to the aglycone at the C-3 position. Thus, the structure of **HC7** was established as methyl 3-*O*-{6-[(2*E*)-*p*-hydroxycinnamoyl]-β-D-glucopyranosyl}-6-methyl-5-hydroxyheptanoate and was given the trivial name hypercerastoside C.

Compounds **HC4** and **HC6** were new natural products, while **HC5** and **HC7** were absent in the ethanol extract analyzed by LC/MS, and should be regarded as artifacts produced during extraction with methanol.

### 3.3. Biological Activities of the Isolated Phenolic Compounds from the Ethyl Acetate Extract of the Aerial Parts of Hypericum cerastoides

#### 3.3.1. Radical-Scavenging Activity of Compounds HC1–HC7

The radical scavenging activity of the isolated compounds **HC1**–**HC7** was evaluated using the DPPH and ABTS assays. (Table 4). The activity of the tested compounds **HC1**–**HC7** (100 µM) was compared to that of Vitamin C and Trolox (positive controls) at the same concentrations, and expressed as a percentage of inhibition against DPPH and ABTS, respectively.

The highest DPPH radical scavenging activity was observed for compounds **HC2** (myricetin-3-O-glucoside) (84.32%), **HC3** (myricetin-3-O-galactoside) (82.70%), and **HC5** (methyl ester of chlorogenic acid) (84.57%). Their activity exceeded that of Vitamin C and was very close to that of Trolox. In contrast, the other compounds—**HC1** (18.95%), **HC4** (14.63%), **HC6** (15.93%), and **HC7** (11.48%)—exhibited low DPPH radical scavenging activity.

Regarding ABTS radical scavenging activity, the results were similar. The myricetin flavonoids, compounds **HC2** (97.23%) and **HC3** (96.04%), exhibited the highest activity. Their activity exceeded that of the positive controls, Vitamin C (66.21%) and Trolox (94.16%). Excellent ABTS radical scavenging activity, higher than that of Vitamin C, was also observed for compound **HC4** (70.20%) (hyperceratoside A) and **HC5** (71.27%) (methyl ester of chlorogenic acid). The remaining compounds—**HC1** (22.12%), **HC6** (25.50%), and **HC7** (26.88%)—showed lower ABTS activity.

#### 3.3.2. α-Glucosidase Inhibitory Activity of Compounds HC1–HC7

The compounds **HC1**–**HC7** were evaluated for their inhibitory activity against α-glucosidase, with acarbose used as a positive control. The IC_50_ values were calculated based on five different concentrations of the test substances and the reference inhibitor (1000 µM, 500 µM, 250 µM, 125 µM, and 62.5 µM). Among the evaluated compounds, **HC1** (coumaroylquinic acid) exhibited the most potent α-glucosidase inhibitory activity, with an IC_50_ of 44 µM, significantly surpassing that of acarbose (IC_50_ = 206 µM). Myricetin-3-O-galactoside (**HC3**) demonstrated an inhibitory effect equivalent to the reference compound (IC_50_ = 206 µM). In contrast, **HC6** (hypercerastoside B) showed lower inhibitory activity, with an IC_50_ value of 371 µM. The remaining compounds—**HC2**, **HC4**, **HC5**, and **HC7**—did not exhibit measurable inhibitory activity under the tested conditions (Table 5).

#### 3.3.3. Lipase Activity of Compounds HC1–HC7

The isolated compounds were evaluated for their modulatory effects on lipase activity at five concentrations (200, 100, 50, 25, and 12.5 µM). Orlistat (IC_50_ = 291.6 µM), marketed as Xenical and widely used in the treatment of obesity, was employed as a negative control. Orlistat functions by inhibiting pancreatic lipase, an enzyme responsible for breaking down dietary fats. By blocking this enzymatic activity, orlistat prevents the hydrolysis and subsequent absorption of triglycerides, resulting in the excretion of undigested fats from the body [39].

The results (Table 6) demonstrate that compounds **HC1** (coumaroylquinic acid), **HC2** (myricetin-3-O-glucoside), **HC3** (myricetin-3-O-galactoside), **HC5** (methyl ester of chlorogenic acid), and **HC6** (hypercerastoside B) do not inhibit lipase activity; instead, they enhance it, exhibiting pro-lipase activity. Among them, the myricetin glycosides (compounds **HC2** and **HC3**) showed the most pronounced effect, increasing lipase activity by approximately fivefold at a concentration of 200 µM compared to the control. Compound **HC5** (methyl ester of chlorogenic acid) also demonstrated notable stimulation, enhancing enzymatic activity by 1.5-fold (169.79%) at the same concentration. In contrast, compounds **HC6** (hypercerastoside B) and **HC1** (coumaroylquinic acid) exhibited the weakest pro-lipase effects, increasing activity by 36.55% and 31.24%, respectively, at 200 µM. Furthermore, compounds **HC4** (hypercerastoside A) and **HC7** (hypercerastoside C) did not show any significant effect on lipase activity at lower concentrations.

## 4. Discussion

A previously developed and validated UHPLC-HRMS method was used for semi-quantitative determination of the identified metabolites in the aerial parts of *Hypericum cerastoides*. The quantity of the identified metabolites was calculated using external standards hyperoside, chlorogenic acid, or mangiferin [18]. The results are given in Table S1. A total of 39 metabolites were tentatively identified. The largest group is the group of flavonol aglycones and their glycosides. It consists of 22 compounds, and their amount was calculated at 2734.24 µg/g D.W. relative to hyperoside. The compounds in the most significant amounts are rutin **18** (470.62 µg/g D.W), isoquercitrin **19** (470.81 µg/g D.W), and myricetin-3-O-galactoside **12** (259.44 µg/g D.W). Except for compounds **18**, **19**, and quercetin **36**, all the remaining flavonoids, including the quercetin glycosides **17**, **22**, **23**, **27**, **29**, **30**, and **34**, the myricetin **33** and its glycosides **12**, **13**, **15**, **16**, **20**, **21**, and **32**, as well as kaempferol **37** and its glucosides **24**, **26**, and 28, are reported here for the first time in *Hypericum cerastoides*. Five compounds represented the group of flavan-3-ols, and their total quantity was 527 µg/g D.W., calculated as hyperoside. Catechin **3** (193.22 µg/g D.W.) and procyanidin B2 **6** (132.22 µg/g D.W.) were found in the highest amounts. Except for epicatechin **9**, all remaining flavan-3-ols, including compounds **1–3** and **6,** are reported here for the first time in *H. cerastoides*. The group of hydroxycinnamic acids consisted of 6 compounds, and their total quantity was calculated to be 367.71 µg/g D.W., expressed as chlorogenic acid. The most significant quantity was 5-O-feruloylquinic acid **10** (136.98 µg/g D.W). All detected hydroxycinnamic acids are reported here for the first time in *H. cerastoides*. Additionally, a flavolignan **31**, a xanthone **35**, two benzophenones **14** and **25**, and two flavone dimers **38** and **39** were found in small amounts. Their amounts ranged from 66.65 to 157.12 µg/g D.W. The compounds **35**, **38**, and **39** are common in *Hypericum* species, while cinchonain Ib **31** is reported here for the first time in this genus.

An extensive chromatographic procedure of the EtOAc extract from the aerial parts of *Hypericum cerastoides* (Spach) N. Robson resulted in the isolation and structural identification of seven phenolic compounds (**HC1**–**HC7**) (Figure 1). Hyperceratoside A (**HC4**) and hyperceratoside B (**HC6**) are new natural products, while methyl ester of chlorogenic acid (**HC5**) and hyperceratoside C (**HC7**) were not detected in the EtOH extract and considered to be artifacts arising from the extraction with methanol. The isolated compounds are tested for radical-scavenging (DPPH and ABTS), α-glucosidase inhibitory, and lipase-modulating activities.

The results from DPPH and ABTS assays (Table 4) show that the myricetin glycosides (compounds **HC2** and **HC3**) and the methyl ester of chlorogenic acid (compound **HC5**) have the highest radical scavenging activity in both assays.

Numerous studies have been conducted to investigate the relationship between the chemical structure and radical scavenging activity of two major groups of phenolic compounds—flavonoids and phenolic acids—as well as their depsides.

It has been established that flavonoids must meet three key structural criteria to exhibit vigorous radical scavenging activity. First, the presence of a 3′,4′-ortho-dihydroxy configuration in the B-ring significantly enhances radical scavenging effectiveness. Second, a C2–C3 double bond configured with a 4-carbonyl group in the C-ring is essential. Third, the presence of a 3-OH group or both 3-OH and 5-OH groups in the A and C rings is also critical for optimal activity [40].

The myricetin glycosides (compounds **HC2** and **HC3**) possess a 3′,4′-ortho-dihydroxyl configuration in the B-ring, a C2–C3 double bond conjugated with a 4-keto group in the C-ring, and hydroxyl groups at positions 5 and 7 in the A-ring—structural features that account for their vigorous radical scavenging activity in both DPPH and ABTS assays. **HC2** and **HC3** exhibit very similar activity levels in both assays. The only structural difference between them lies in the sugar moiety attached at position 3 of the C-ring—glucose in **HC2** and galactose in **HC3**—which does not appear to influence their radical scavenging potential significantly.

The radical scavenging activity of hydroxycinnamic acids and their depsides depends mainly on the number and position of hydroxyl groups. Methoxylation of hydroxyl groups typically reduces the radical scavenging capacity of these compounds [40]. The methyl ester of chlorogenic acid (**HC5**) is a caffeic depside with quinic acid. Its high radical scavenging activity—84.57% in the DPPH assay and 71.27% in the ABTS assay—is likely attributed to the presence of five hydroxyl groups: two in the caffeic acid moiety and three in the quinic acid.

The results from the α-glucosidase inhibitory assay (Table 5) indicate that compounds **HC1**, **HC3**, and **HC6** exhibit inhibitory effects comparable to those of acarbose (marketed as Glucobay), a drug commonly used to prevent type 2 diabetes in individuals with impaired glucose tolerance. It is well established that acarbose delays the breakdown of carbohydrates by inhibiting α-glucosidase, an intestinal enzyme responsible for the hydrolysis of complex carbohydrates such as starch and sucrose into glucose. By inhibiting this enzyme, acarbose slows down the release of glucose into the bloodstream, thereby attenuating postprandial blood glucose spikes. This mechanism contributes to the reduction in daily glycemic fluctuations and leads to lower average blood glucose levels. The findings suggest that *Hypericum cerastoides* represents a promising natural source for the isolation of compounds with α-glucosidase inhibitory activity.

The results for the lipase-modulating activity test (Table 6) demonstrate that compounds **HC1**, **HC2**, **HC3**, **HC5**, and **HC6** exhibit pro-lipase activity. The myricetin glycosides (**HC2** and **HC3**) and methyl ester of chlorogenic acid (**HC5**) are the most active constituents, while **HC6** and **HC1** exhibited the weakest pro-lipase effects. Compounds exhibiting pro-lipase activity have been identified as potential therapeutic agents for the treatment of cachexia [41]. Cachexia is a pathological condition characterized by severe body wasting, marked by significant weight loss accompanied by the depletion of both adipose tissue and skeletal muscle mass. This syndrome is most commonly observed in patients with advanced-stage cancer, as well as in individuals suffering from anorexia or severe infectious diseases [42].

## 5. Conclusions

A UHPLC-HRMS-based profiling of hydroalcoholic extract from the aerial parts of *Hypericum cerastoides* (Spach) N. Robson tentatively identified 39 phenolic compounds (flavonoids, flavan-3-ols, and a flavolignan, as well as hydrohycinnamic acid derivatives, benzophenones, a xanthone, and flavone dimers). The main groups were flavonoids (22 compounds) and hydroxycinnamic acid derivatives (6 compounds), and their total amount was calculated at 2734.24 and 527 µg/g D.W. relative to hyperoside, respectively. Those present in the most significant quantities were rutin **18** (470.62 µg/g D.W), isoquercitrin **19** (470.81 µg/g D.W), myricetin-3-O-galactoside **12** (259.44 µg/g D.W), and 5-O-feruloylquinic acid **10** (136.98 µg/g D.W).

Two new phenolic compounds, namely hypercerastoside A (**HC4**) and hypercerastoside B (**HC6**), together with three known compounds, coumaroylquinic acid (HC1), myricetin-3-O-glycoside (**HC2**), and myricetin-3-O-galactoside (**HC3**), as well as two artifacts, namely methyl ester of chlorogenic acid (**HC5**) and hypercerastoside C (**HC7**), were isolated from the ethylacetate extract of the aerial parts of title plant and identified using spectroscopic methods (1D and 2D NMR, UV, and HRMS-ESI). Isolated compounds were tested for radical-scavenging, anti-α-glucosidase, and pro-lipase activity.

The radical scavenging activity of the isolated compounds **HC1**–**HC7** was evaluated using both the DPPH and ABTS assays. The results revealed that the myricetin glycosides (compounds **HC2** and **HC3**), along with the methyl ester of chlorogenic acid (compound **HC5**), exhibited the highest radical scavenging activity in both assays. Their activity was either comparable to or exceeded that of standard antioxidants such as vitamin C and Trolox.

Compound **HC1** (coumaroylquinic acid) demonstrated the most vigorous α-glucosidase inhibitory activity, with an IC_50_ value of 44 µM, significantly surpassing that of acarbose (IC_50_ = 206 µM). Myricetin-3-O-galactoside (**HC3**) exhibited inhibitory activity equivalent to that of the reference compound (IC_50_ = 206 µM). These findings suggest that compounds **HC1** and **HC3** exert effects comparable to acarbose, a clinically used α-glucosidase inhibitor for the prevention of type 2 diabetes in individuals with impaired glucose tolerance.

The myricetin glycosides (compounds **HC2** and **HC3**) exhibited the highest pro-lipase activity, enhancing lipase enzyme function by approximately fivefold at a concentration of 200 µM compared to the control. This pronounced stimulatory effect may suggest their potential application in the treatment of cachexia, a condition commonly associated with cancer, anorexia, and infectious diseases.

Overall, the findings suggest that *H. cerastoides* is a promising source of bioactive phenolic compounds with notable radical-scavenging and enzyme-modulating activities. These results emphasize its potential therapeutic relevance, especially for oxidative stress-related disorders, type 2 diabetes, and cachexia, and support further research to validate and expand these findings. The LC-MS profiling performed in this study offers a strong basis for future detailed phytochemical research focused on isolating a wider variety of biologically active compounds and validating their activity through in vivo experiments.

## Figures and Tables

**Table 1:** ^1^H and ^13^C NMR spectral data (in ppm) of compound **HC4**.

Position	*δ*_H_, Int., Mult. (*J* in Hz)	*δ*_C_ (ppm), Mult. ^1^
1	-	109.4, C
2	-	158.6, C
3	6.32, 1H, d (2.0)	95.8, CH
4	-	162.4, C
5	6.18, 1H, d (2.0)	98.0, CH
6	-	160.0, C
1′	-	140.3, C
2′	7.72, 1H, m	129.9, CH
3′	7.44, 1H, m	128.9, CH
4′	7.55, 1H, m	133.1, CH
5′	7.44, 1H, m	128.9, CH
6′	7.72, 1H, m	129.9, CH
C=O	-	196.5, C
1″	5.01, 1H, d (8.0)	99.6, CH
2″	4.46, 1H, dd (8.0, 9.6)	73.7, CH
3″	3.55, 1H, dd (9.6, 9.0)	75.9, CH
4″	3.41, 1H, dd (8.9, 9.6)	71.3, CH
5″	3.50, 1H, m	77.9, CH
6″	3.90, 1H, dd (2.6, 11.9) 3.71, 1H, dd (5.6, 11.9)	62.4, CH_2_
1‴	-	169.3, C
2‴	1.78, 3H, s	20.7, CH_3_

^1^ The ^1^H-NMR spectrum and HSQC experiment determined multiplicities.

**Table 2:** ^1^H (at 600 MHz) and ^13^C NMR (at 150 MHz) spectral data (in ppm) of compound **HC6** taken in (CD_3_)_2_CO.

Position	*δ*_H_, Int., Mult. (*J* in Hz)	*δ*_C_ (ppm), Mult. ^1^
2	-	169.8, C
3	2.67, 2H, d (3.8)	37.0, CH_2_
4	4.35, 1H, m	72.9, CH
5	2.27, 1H, m; 1.74, 1H, m	31.7, CH_2_
6	4.45, 1H, m	80.9, CH
7	1.80, 1H, m	33.1, CH
8	0.91, 2H, d (6.8)	18.4, CH_3_
9	0.91, 2H, d (6.8)	17.7, CH_3_
1′	4.50, 1H, d (7.8)	104.2, CH
2′	3.24, 1H, dd (9.0, 7.8)	74.7, CH
3′	3.45, 1H, dd (9.0, 8.8)	77.6, CH
4′	3.40, 1H, dd (9.2, 8.8)	71.2, CH
5′	3.61, 1H, m	75.0, CH
6′	4.52, 1H, dd (11.8, 2.0); 4.29, 1H, dd (11.8, 6.4)	64.3, CH_2_
1″	-	167.5, C
2″	6.39, 1H, d (16.0)	115.3, CH
3″	7.64, 1H, d (16.0)	145.6, CH
4″		126.9, C
5″	7.57, 2H, d (8.6)	131.0, CH
6″	6.91, 2H, d (8.6)	116.7, CH
7″		160.7, CH
8″	6.91, 2H, d (8.6)	116.7, CH
9″	7.57, 2H, d (8.6)	131.0, CH

^1^ The ^1^H-NMR spectrum and HSQC experiment determined multiplicities.

**Table 3:** ^1^H (at 600 MHz) and ^13^C NMR (at 150 MHz) spectral data (in ppm) of compound **HC7** taken in (CD_3_)_2_CO.

Position	*δ*_H_, Int., Mult. (*J* in Hz)	*δ*_C_ (ppm), Mult. ^1^
1	-	172.8, C
2	2.65, 2H, m	40.2, CH_2_
3	4.30, 1H, m	76.8, CH
4	1.78, 1H, m; 1.69, 1H, m	40.3, CH_2_
5	3.54, 1H, m	73.8, CH
6	1.64, 1H, m	34.4, CH
7	0.85, 3H, d (6.8)	19.3, CH_3_
8	0.83, 3H, d (6.8)	17.4, CH_3_
9	3.64, 3H, s	51.8, CH_3_
1′	4.49, 1H, d (7.8)	103.9, CH
2′	3.18, 1H, dd (9.0, 8.8)	74.9, CH
3’	3.45, 1H, dd (9.0, 8.8)	77.7, CH
4’	3.37, 1H, dd (9.0, 8.8)	71.4, CH
5’	3.61, 1H, m	75.0, CH
6’	4.52, 1H, dd (11.8, 2.2) 4.27, 1H, dd (11.8, 6.6)	64.5, CH_2_
1’’	-	167.5, C
2’’	6.40, 1H, d (16.0)	116.7, CH
3’’	7.63, 1H, d (16.0)	145.5, CH
4″	-	127.0, C
5″	7.57, 2H, d (8.6)	131.0, CH
6″	6.91, 2H, d (8.6)	115.5, CH
7″	-	160.6, C
8″	6.91, 2H, d (8.6)	116.7, CH
9″	7.57, 2H, d (8.6)	131.0, CH

^1^ The HSQC experiment determined multiplicities.

**Table 4:** DPPH and ABTS radical-scavenging activity of compounds **HC1**–**HC7**.

Compounds	DPPH % ^1^	ABTS % ^2^
coumaroylquinic acid (**HC1**)	18.95 ± 0.36	22.12 ± 0.63
myricetin-3-O-glucoside (**HC2**)	84.32 ± 0.23	97.23 ± 0.45
myricetin-3-O-galactoside (**HC3**)	82.70 ± 0.34	96.04 ± 0.37
hypercerastoside A (**HC4**)	14.63 ± 0.50	70.20 ± 0.23
methyl ester of chlorogenic acid (**HC5**)	84.57 ± 0.27	71.27 ± 0.08
hypercerastoside B (**HC6**)	15.93 ± 0.33	25.50 ± 0.12
hypercerastoside C (**HC7**)	11.48 ± 0.21	26.88 ± 0.33
Vit C	59.44 ± 0.42	66.21 ± 0.45
Trolox	88.33 ± 0.33	94.16 ± 0.32

^1^ *p* < 0.05 vs. control (untreated DPPH). ^2^ *p* < 0.05 vs. control (untreated ABTS).

**Table 5:** *α*-Glucosidase inhibitory activity (IC_50_) of compounds **HC1**–**HC7**.

Compounds	α-Glucosidase Inhibitory Activity (IC_50_ ± SD) ^1^
coumaroylquinic acid (**HC1**)	44 ± 5 µM
myricetin-3-O-glucoside (**HC2**)	NA ^2^
myricetin-3-O-galactoside (**HC3**)	206 ± 13 µM
hypercerastoside A (**HC4**)	NA ^2^
methyl ester of chlorogenic acid (**HC5**)	NA ^2^
hypercerastoside B (**HC6**)	371 ± 9 µM
hypercerastoside C (**HC7**)	NA ^2^
Acarbose	206 ± 10 µM

^1^ *p* > 0.05 vs. control (substrate + untreated enzyme). ^2^ The compound is not active.

**Table 6:** Pro-lipase activity of compounds **HC1**–**HC7**.

Compounds	Pro-Lipase Activity (%) ^1^ at				
	200 µM	100 µM	50 µM	25 µM	12.5 µM
**HC1**	31.24 ± 0.70	24.44 ± 0.46	15.05 ± 0.27	10.86 ± 0.03	7.15 ± 0.17
**HC2**	479.69 ± 7.63	257.38 ± 3.68	137.99 ± 2.69	72.56 ± 1.25	61.94 ± 0.44
**HC3**	492.57 ± 10.25	263.68 ± 4.96	153.47 ± 3.56	85.85 ± 1.19	46.98 ± 0.48
**HC4**	NA ^2^	NA ^2^	NA ^2^	NA ^2^	NA ^2^
**HC5**	169.79 ± 4.23	90.09 ± 2.19	46.87 ± 1.12	28.24 ± 0.61	21.39 ± 0.21
**HC6**	36.55 ± 0.57	27 ± 0.50	15.96 ± 0.23	13.75 ± 0.31	8.28 ± 0.17
**HC7**	NA ^1^	NA ^1^	NA ^1^	NA ^1^	NA ^1^

^1^ *p* > 0.05 vs. control (substrate + untreated enzyme). ^2^ The compound is not active.

## Data Availability

Data are contained within the article and in Supplementary Materials.

## Supplementary Materials

The following supporting information can be downloaded at: https://www.mdpi.com/article/10.3390/metabo15100643/s1, Table S1: The detected and identified polar phenolic compounds and their quantity in the EtOH extract from the aerial parts of *H. cerastoides*; Figures S1–S21: MS and NMR spectra of compounds **HC4**, **HC6**, and **HC7**. Figures S22–S27: A plausible MS/MS fragmentation pattern of some metabolites found in the aerial parts of *H. cerastoides*.
