# Supplementary material for: Phenolic Compounds from Hypericum cerastoides (Spach) N. Robson: Dereplication via UHPLC-HRMS/MS, Isolation, Identification, and Preliminary Biological Evaluation Focusing on Radical-Scavenging, Anti-α-Glucosidase, and Pro-Lipase Activities
Source: Metabolites. 2025 Sep 25;15(10):643. doi: 10.3390/metabo15100643 (PMC12565843; doi:10.3390/metabo15100643)
Supplement: Supplementary file 1 [file metabolites-15-00643-s001.zip › Figures_S1_S21.pdf]

Supplementary Materials

Phenolic compounds from *Hypericum cerastoides* (Spach) N. Robson: dereplication via UHPLC-HRMS/MS, isolation, identification, and preliminary biological evaluation focusing on radical-scavenging, anti-α-glucosidase, and pro-lipase activities.

Zlatina Kokanova-Nedialkova [1], Yana Ilieva [2], Teodor Marinov [1], and Paraskev T. Nedialkov [1,*]

Department of Pharmacognosy, Faculty of Pharmacy, Medical University of Sofia, 2 Dunav Str., 1000 Sofia, Bulgaria; pnedialkov@pharmfac.mu-sofia.bg (P.T.N.), zlatina.kokanova@pharmfac.mu-sofia.bg (Z.K.-N.), t.marinov@pharmfac.mu-sofia.bg (T.M.)

[2] Department of Infectious Microbiology, The Stephan Angeloff Institute of Microbiology, Bulgarian Academy of Sciences, 26 Acad. G. Bonchev Str., 1113 Sofia, Bulgaria; illievayana@gmail.com (Y.I.)

* Correspondence: pnedialkov@pharmfac.mu-sofia.bg

Contents

Figure S1. Full MS spectrum of compound **HC4** in negative mode. 2

Figure S2. MS/MS spectrum of the deprotonated molecule $[M-H]^-$ of **HC4**. 2

Figure S3. ^{1}H-NMR spectrum of compound **HC4**. 3

Figure S4. ^{13}C-NMR spectrum of compound **HC4**. 3

Figure S5. COSY spectrum of compound **HC4**. 4

Figure S6. HSQC spectrum of compound **HC4**. 4

Figure S7. HMBC spectrum of compound **HC4**. 5

Figure S8. Full MS spectrum of compound **HC6** in positive mode. 5

Figure S9. MS/MS spectrum of the protonated molecule $[M+H]^+$ of **HC6**. 6

Figure S10. ^{1}H-NMR spectrum of compound **HC6**. 6

Figure S11. ^{13}C NMR spectrum of compound **HC6**. 7

Figure S12. COSY spectrum of compound **HC6**. 7

Figure S13. HSQC spectrum of compound **HC6**. 8

Figure S14. HMBC spectrum of compound **HC6**. 8

Figure S15. Full MS spectrum of compound **HC7** in positive mode. 9

Figure S16. MS/MS spectrum of the protonated molecule $[M+H]^+$ of **HC7**. 9

Figure S17. ^{1}H NMR spectrum of compound **HC7**. 10

Figure S18. ^{13}C NMR spectrum of compound **HC7**. 10

Figure S19. COSY spectrum of compound **HC7**. 11

Figure S20. HSQC spectrum of compound **HC7**. 11

Figure S21. HMBC spectrum of compound **HC7**. 12

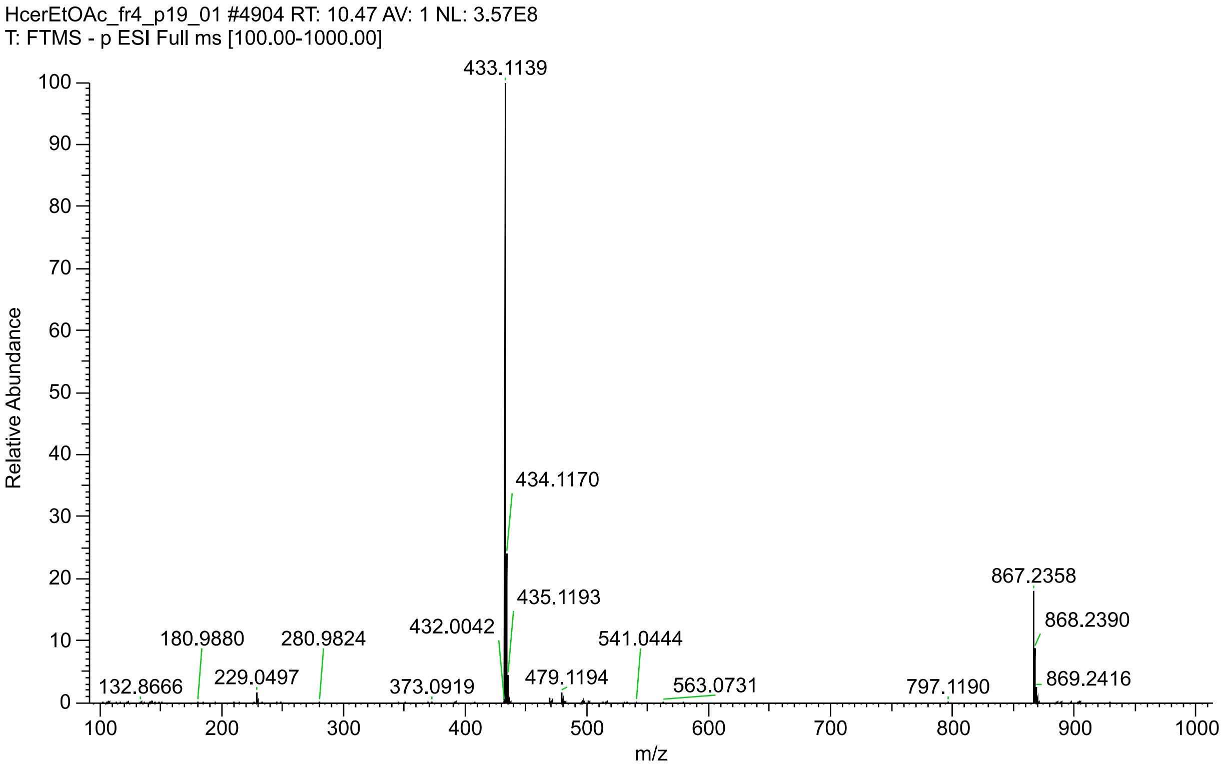

Figure S1. Full MS spectrum of compound **HC4** in negative mode.

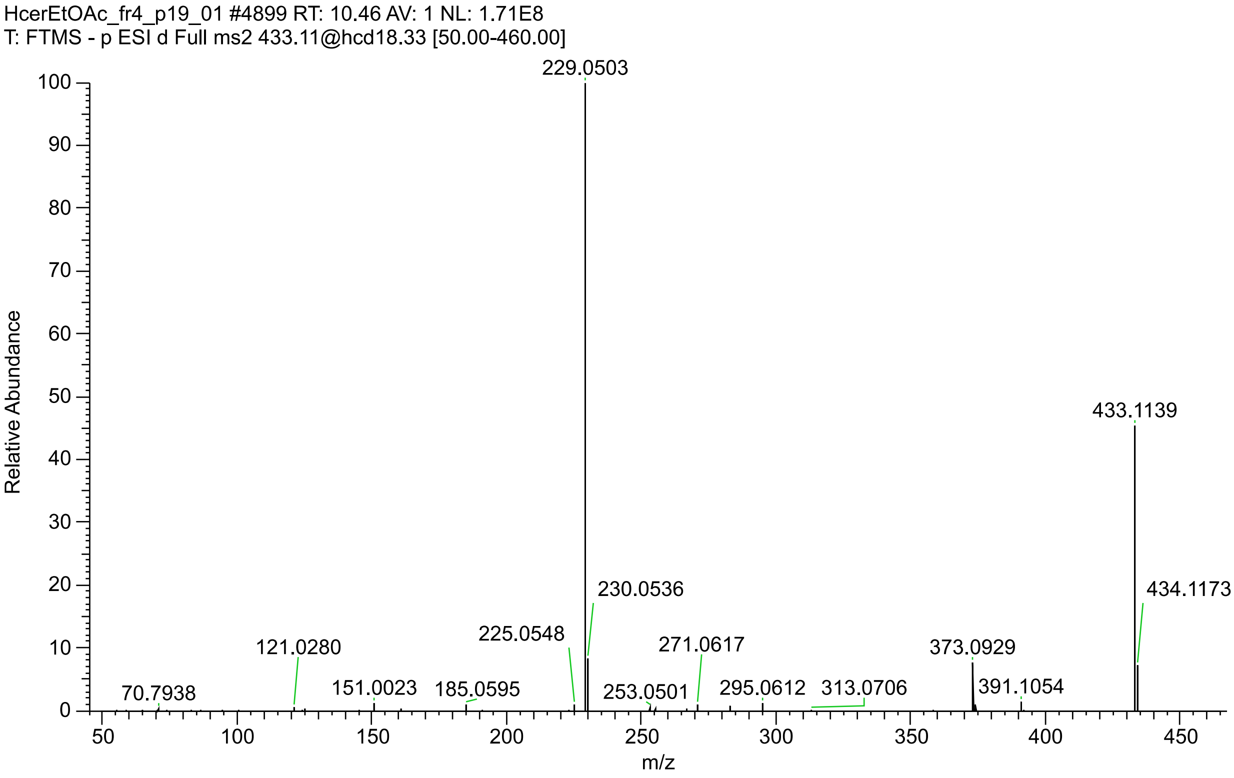

Figure S2. MS/MS spectrum of the deprotonated molecule [M−H]⁻ of **HC4**.

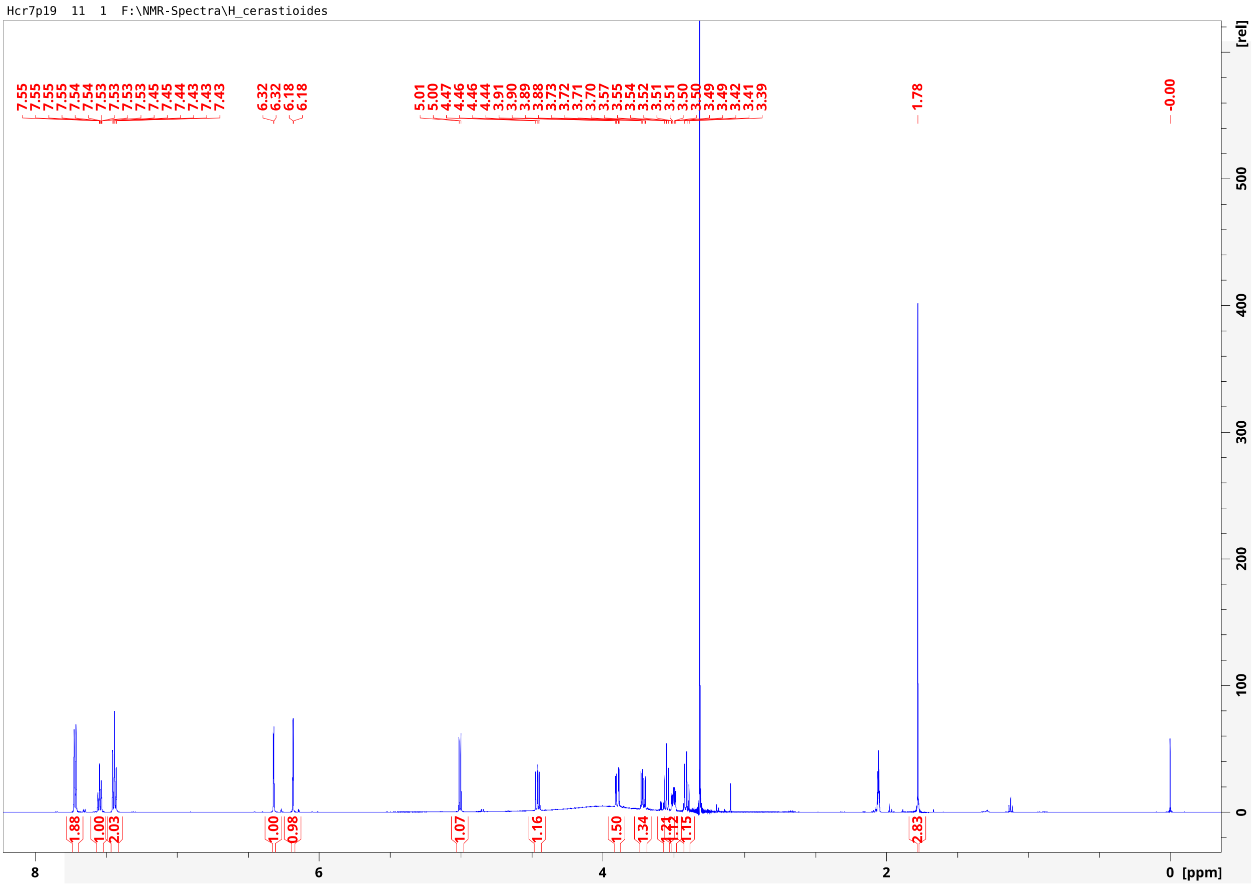

Figure S3. ^{1}H-NMR spectrum of compound **HC4**.

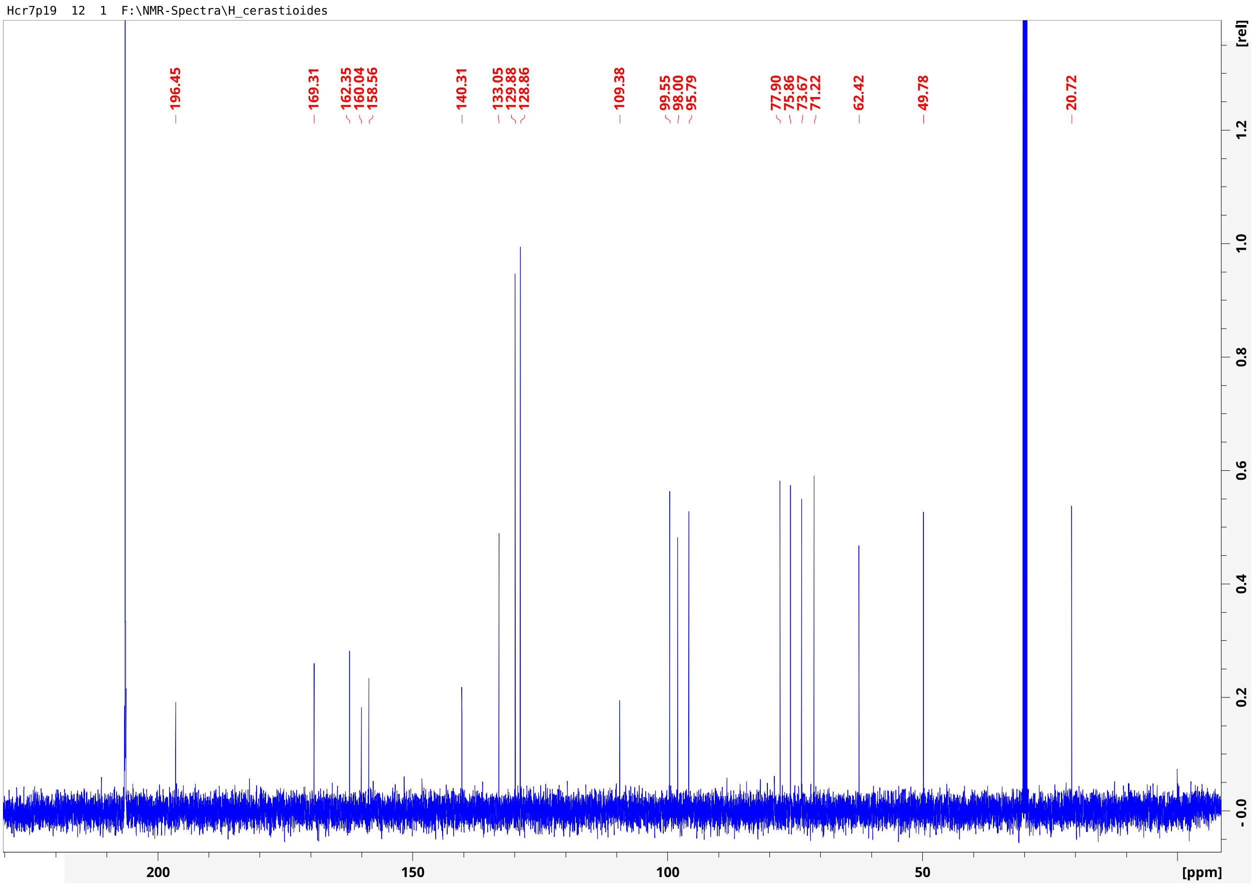

Figure S4. ^{13}C-NMR spectrum of compound **HC4**.

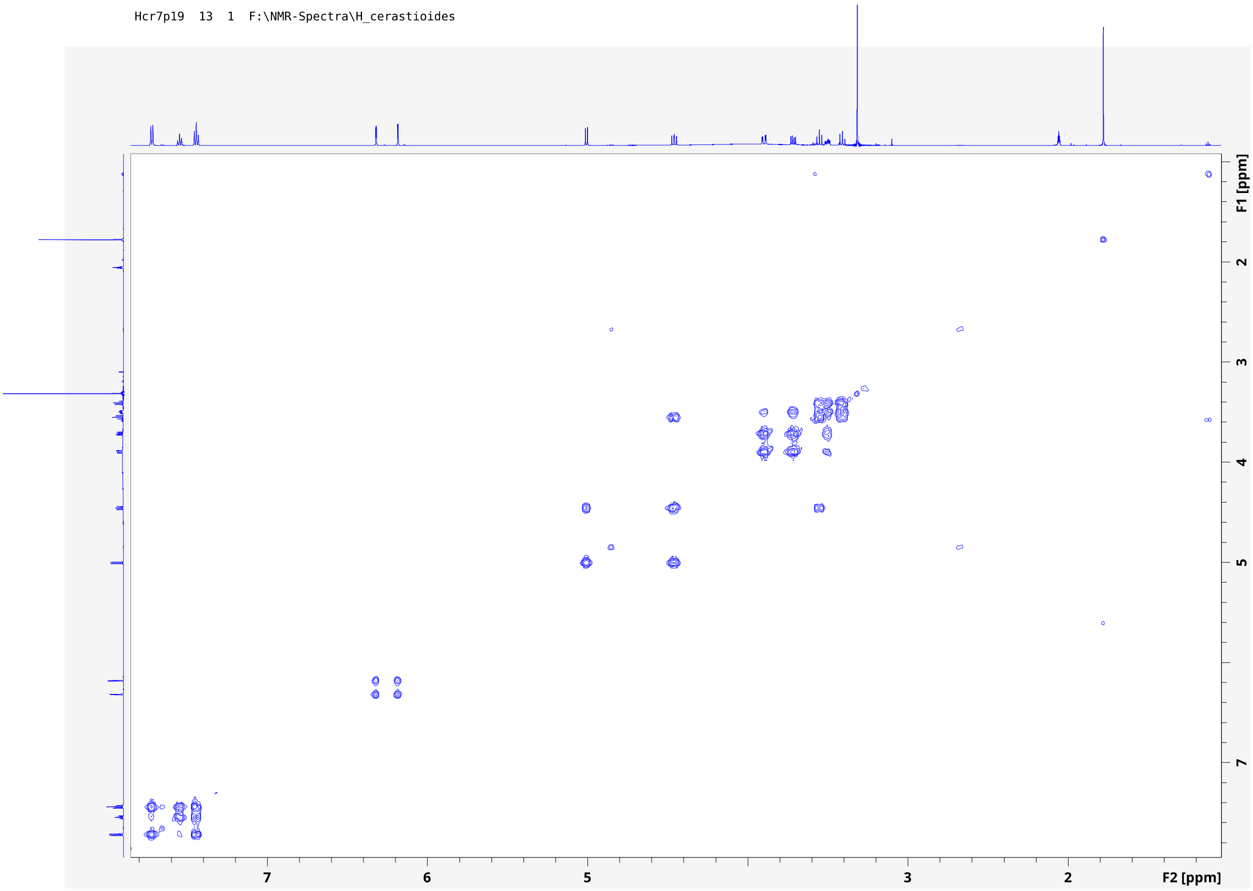

Figure S5. COSY spectrum of compound **HC4**.

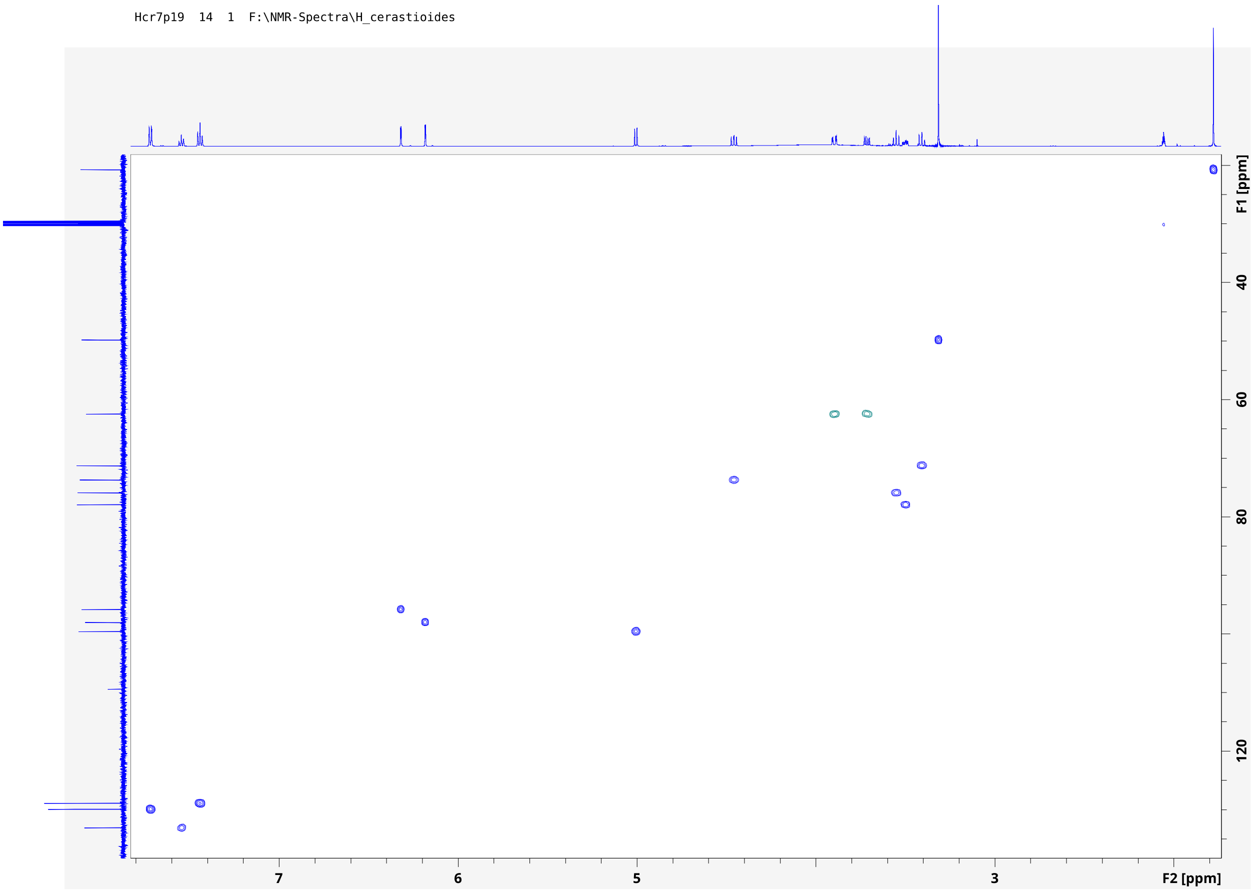

Figure S6. HSQC spectrum of compound **HC4**.

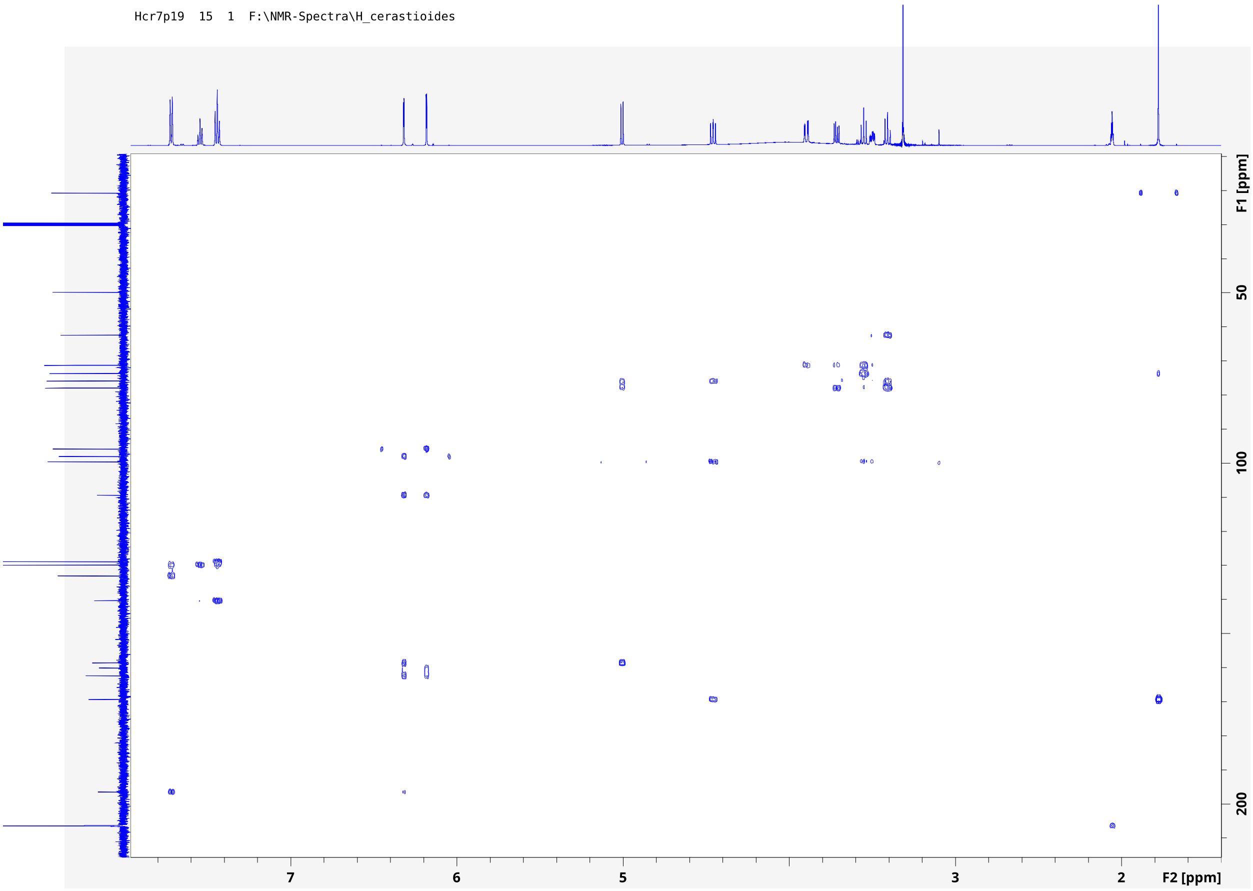

Figure S7. HMBC spectrum of compound **HC4**.

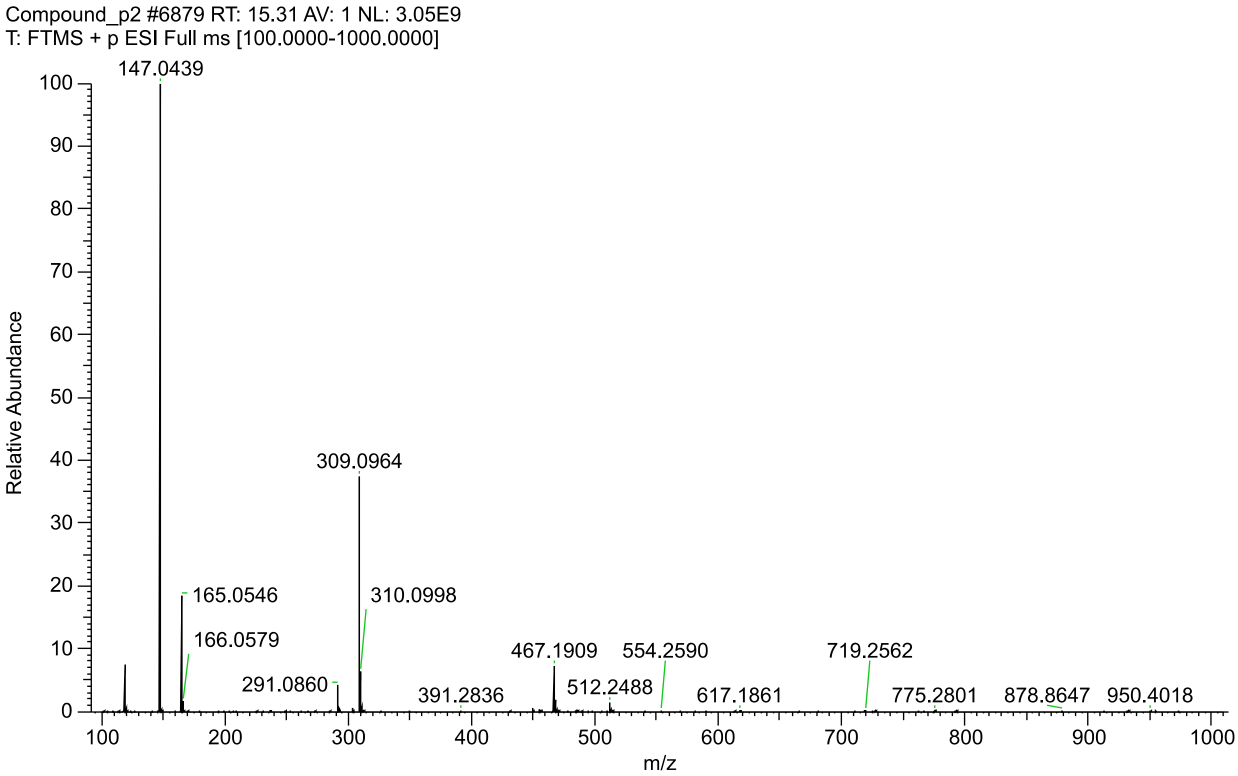

Figure S8. Full MS spectrum of compound **HC6** in positive mode.

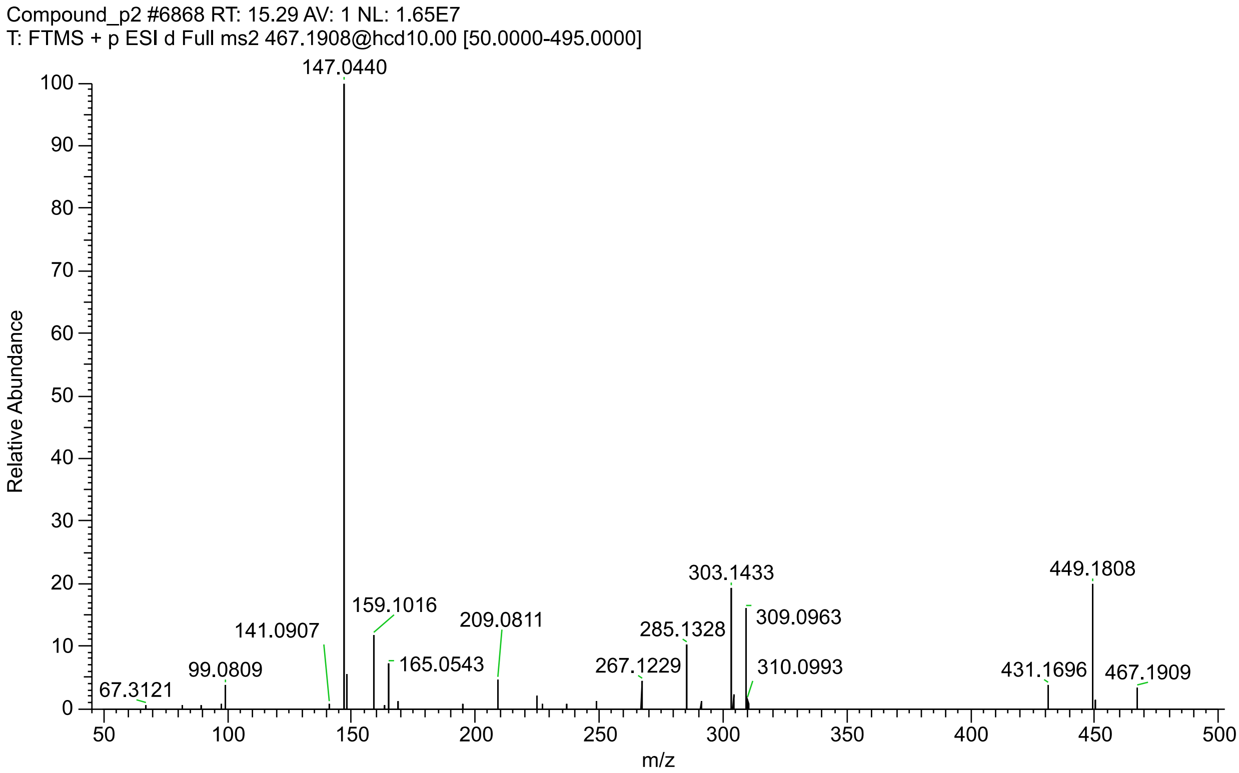

Figure S9. MS/MS spectrum of the protonated molecule $[M+H]^+$ of **HC6**.

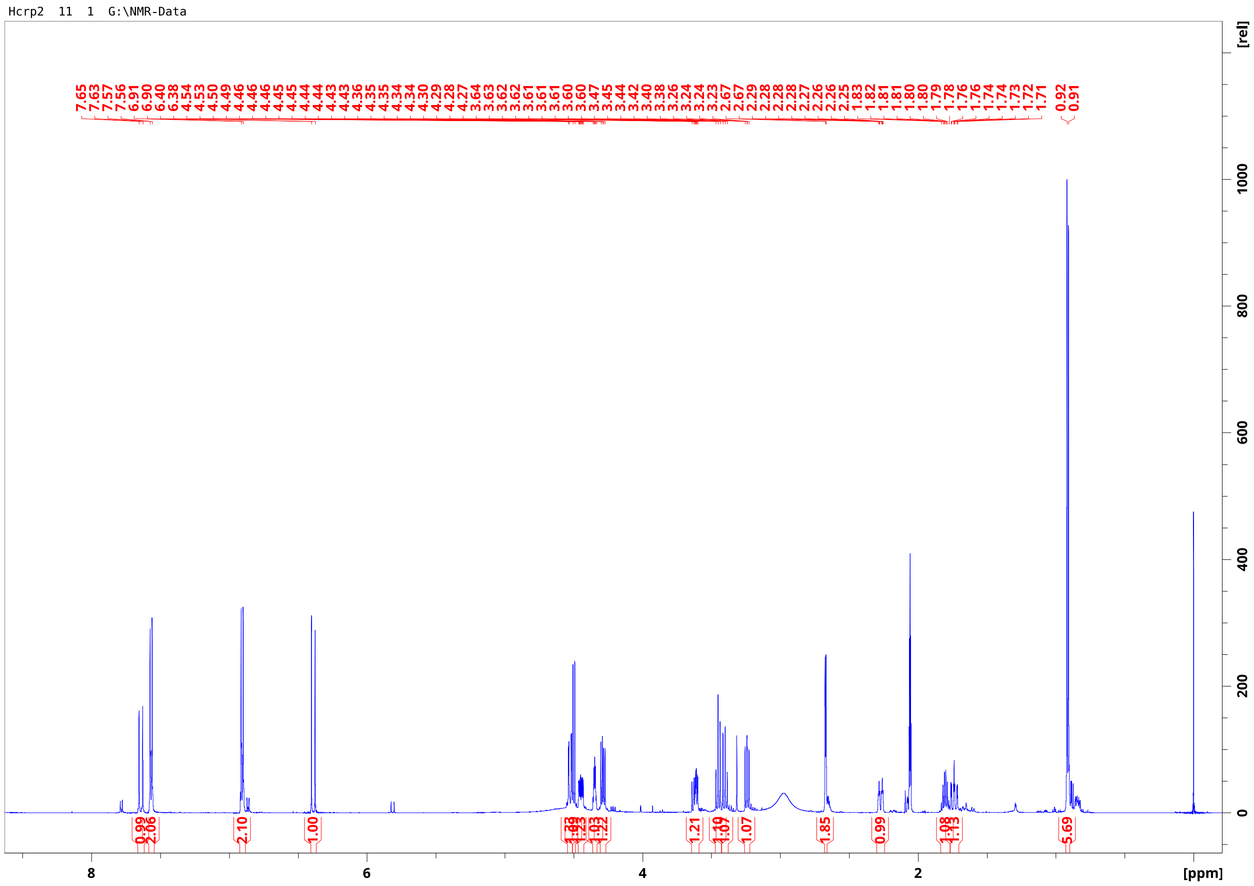

Figure S10. ^{1}H-NMR spectrum of compound **HC6**.

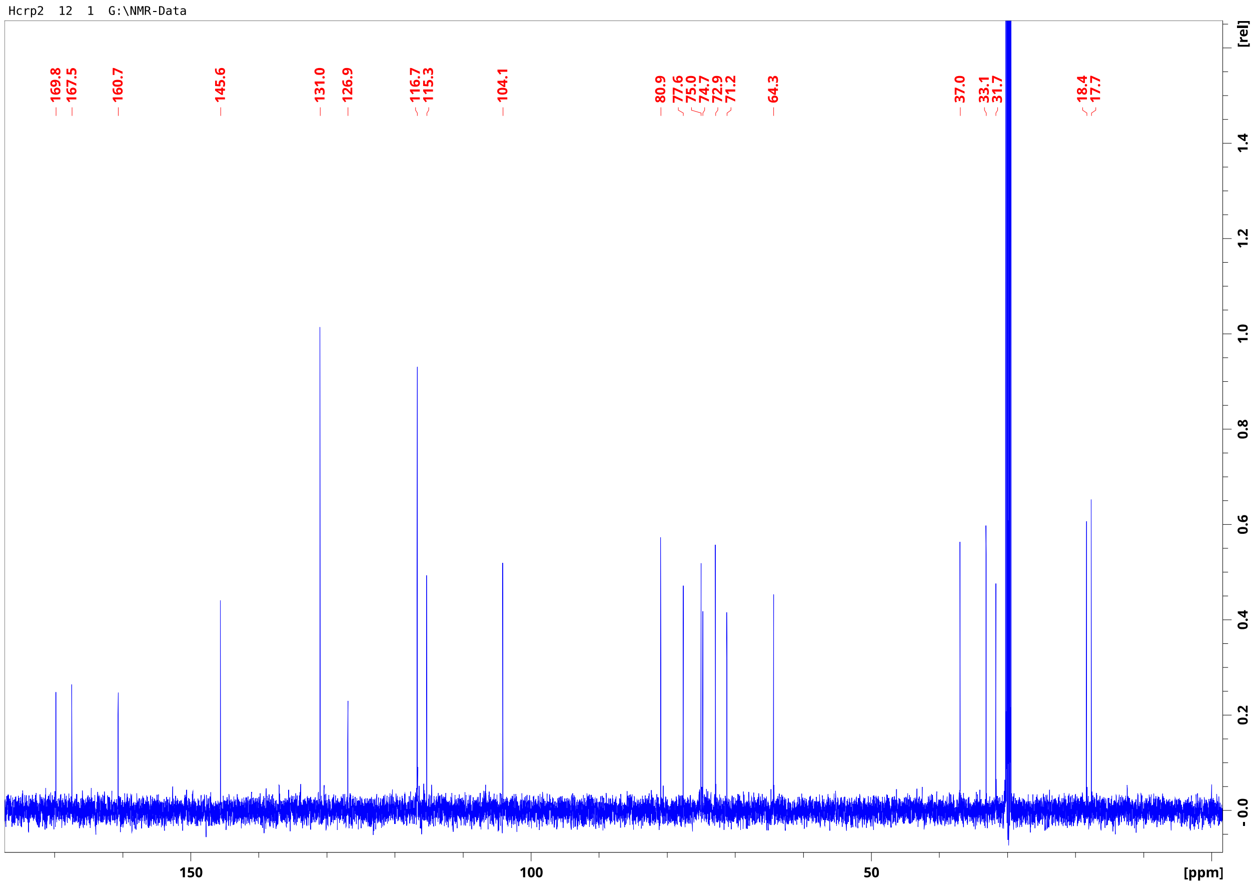

Figure S11. ^{13}C NMR spectrum of compound **HC6**.

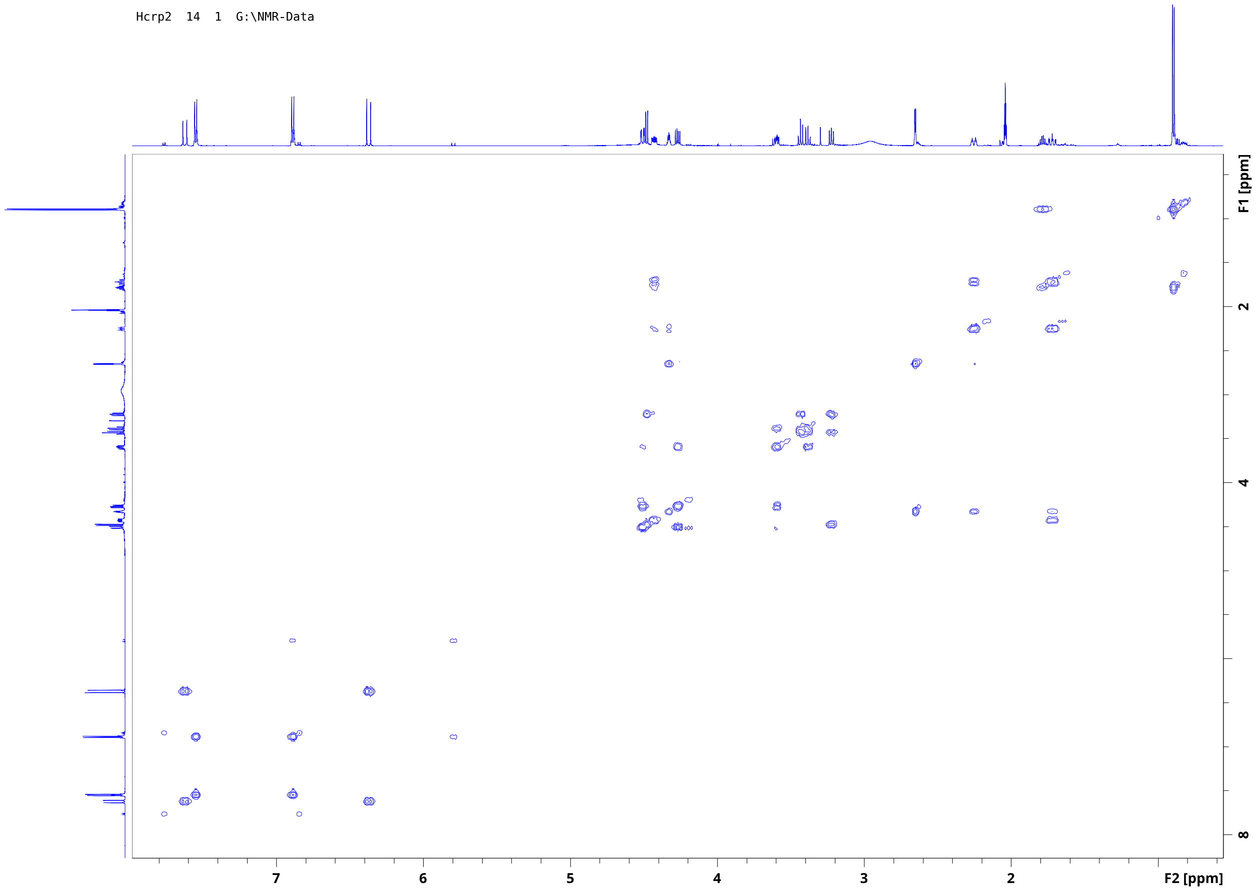

Figure S12. COSY spectrum of compound **HC6**.

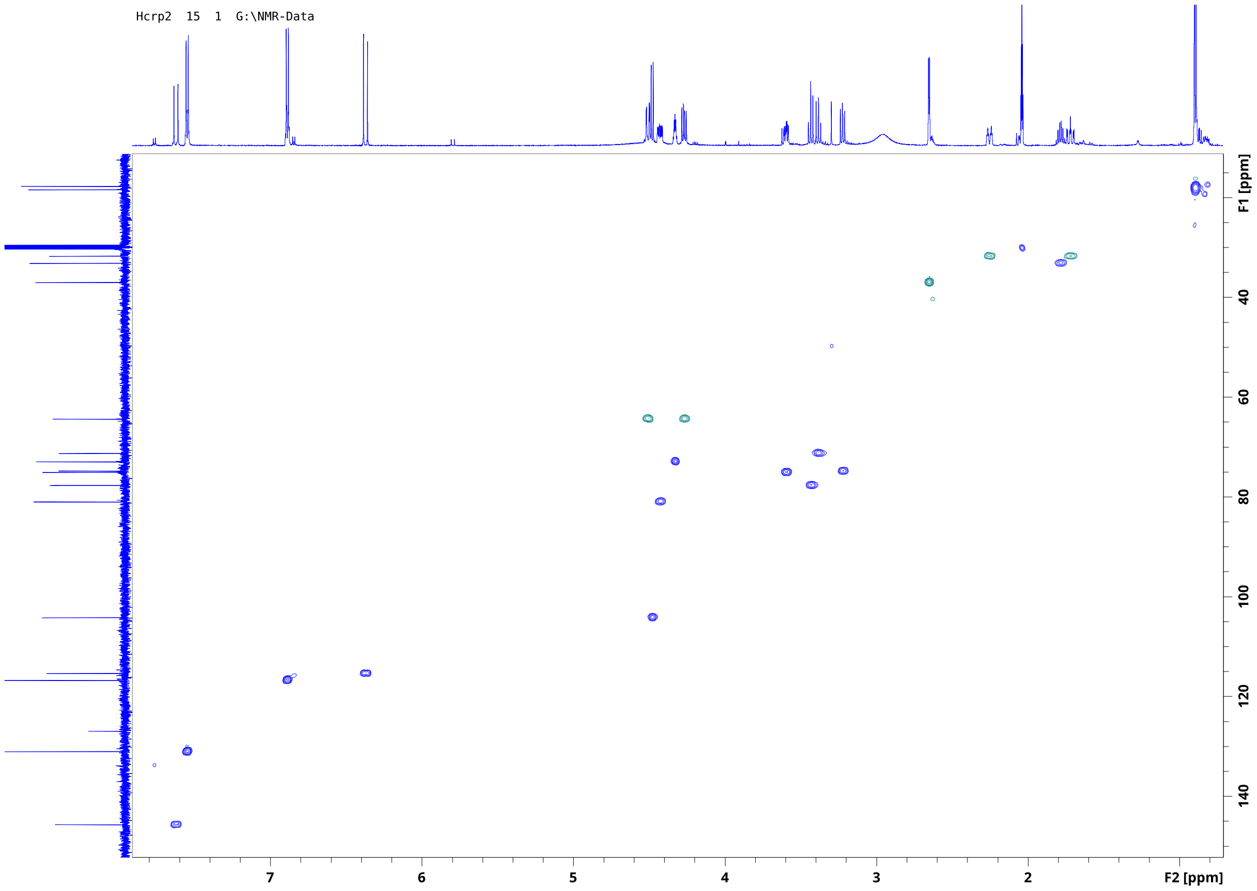

Figure S13. HSQC spectrum of compound **HC6**.

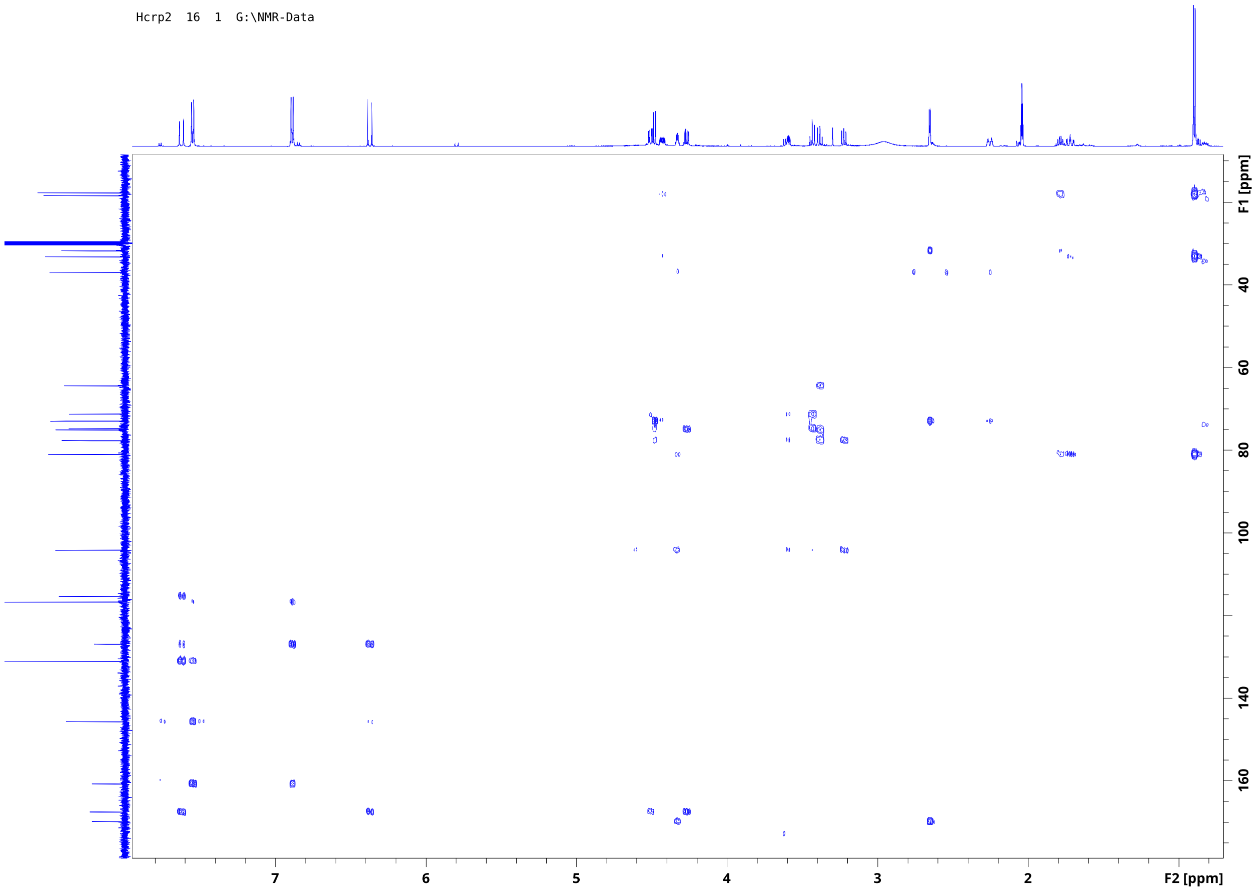

Figure S14. HMBC spectrum of compound **HC6**.

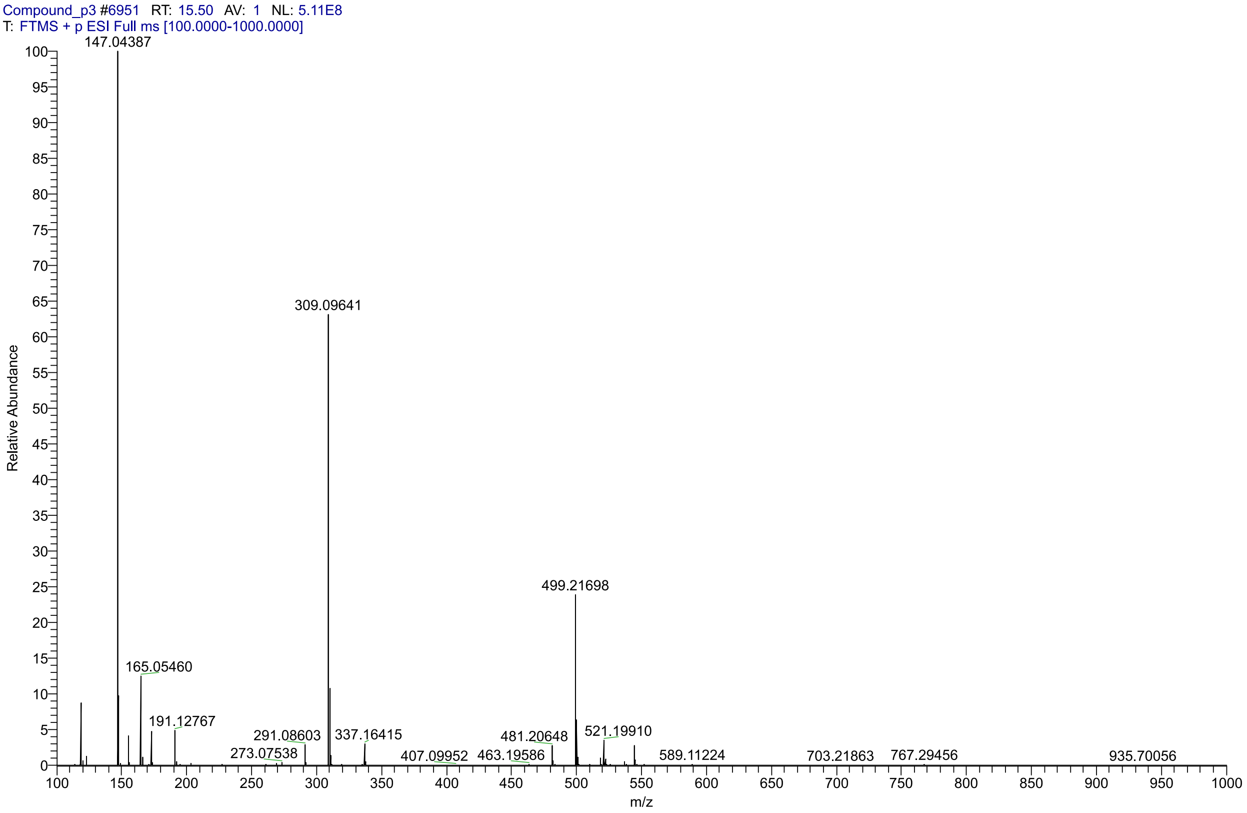

Figure S15. Full MS spectrum of compound **HC7** in positive mode.

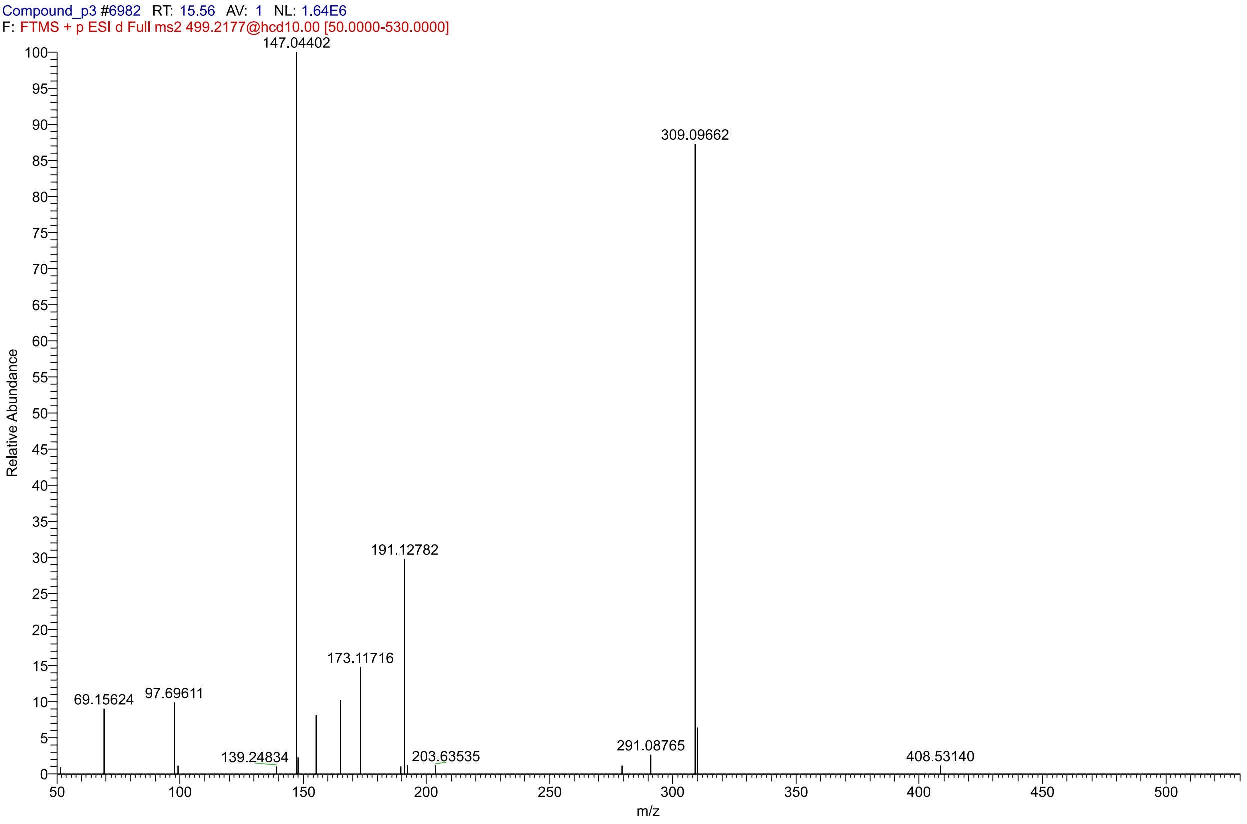

Figure S16. MS/MS spectrum of the protonated molecule $[M+H]^+$ of **HC7**.

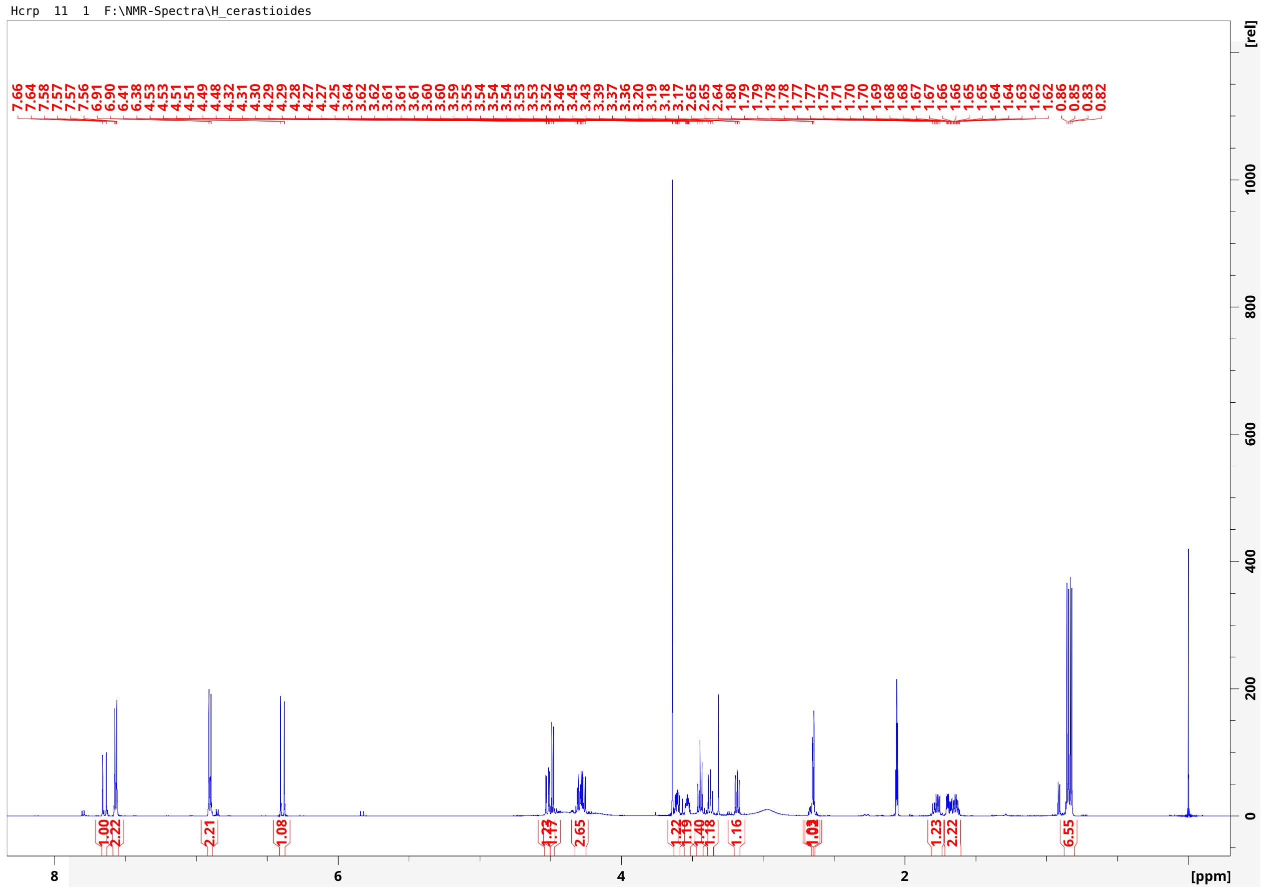

Figure S17. ^{1}H NMR spectrum of compound **HC7**.

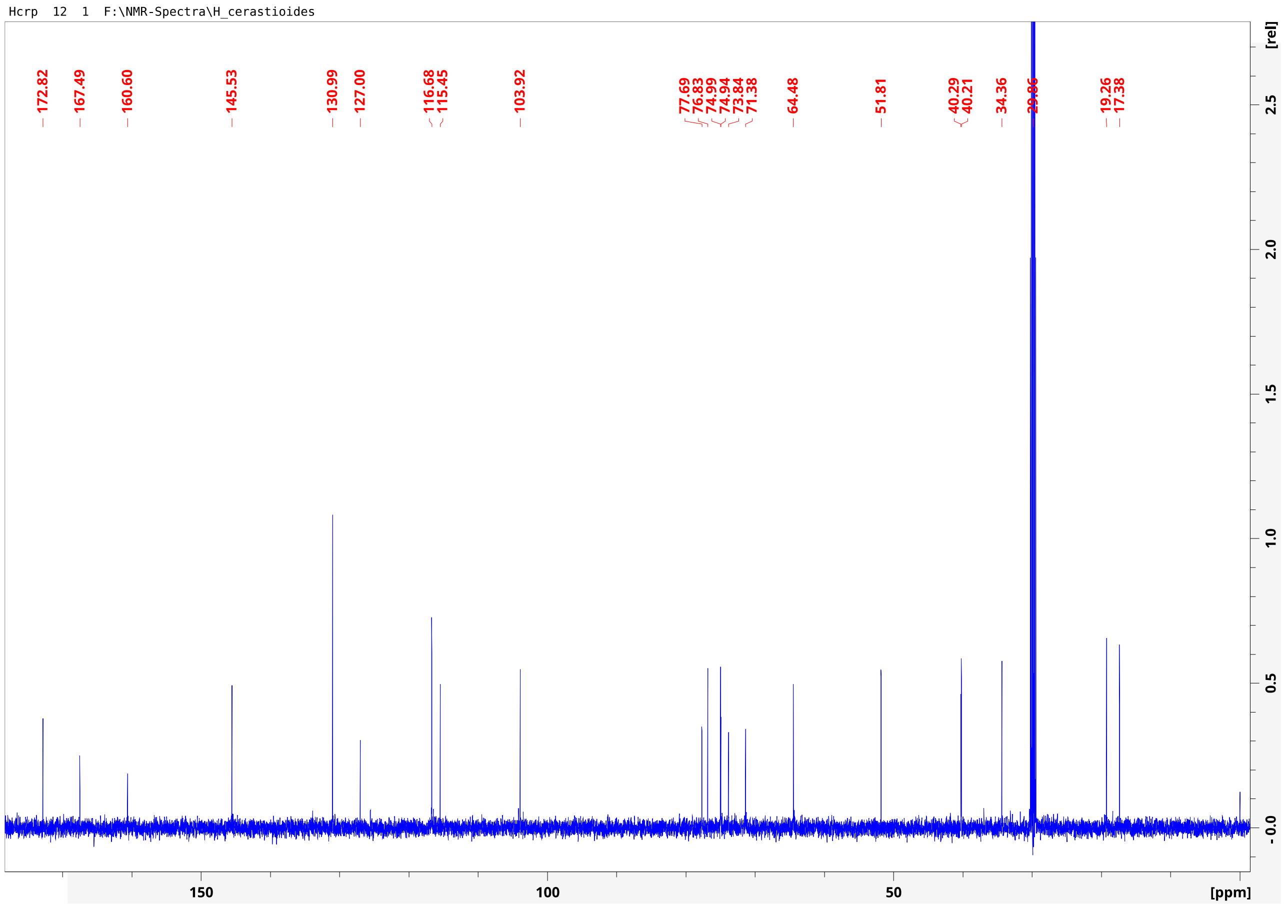

Figure S18. ^{13}C NMR spectrum of compound **HC7**.

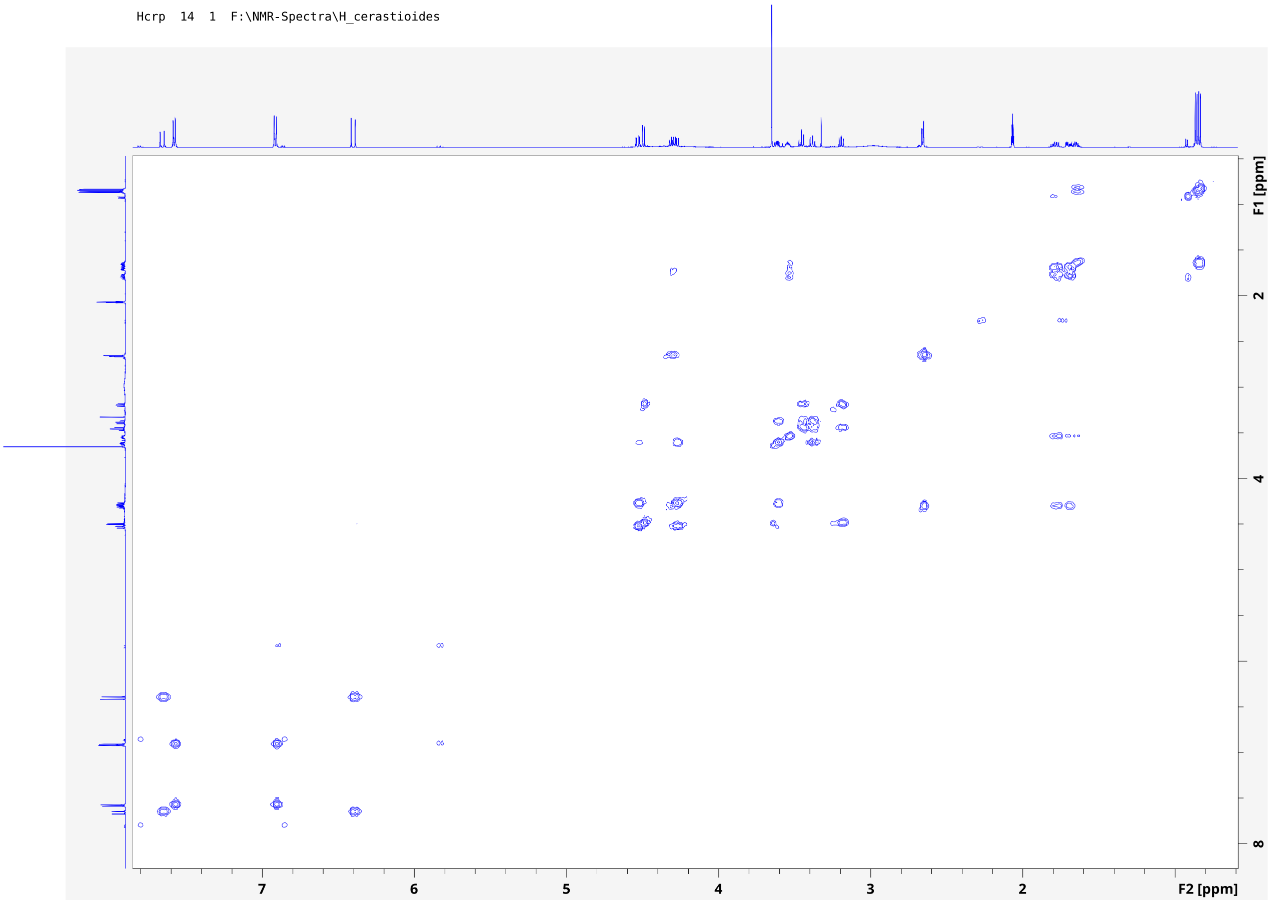

Figure S19. COSY spectrum of compound **HC7**.

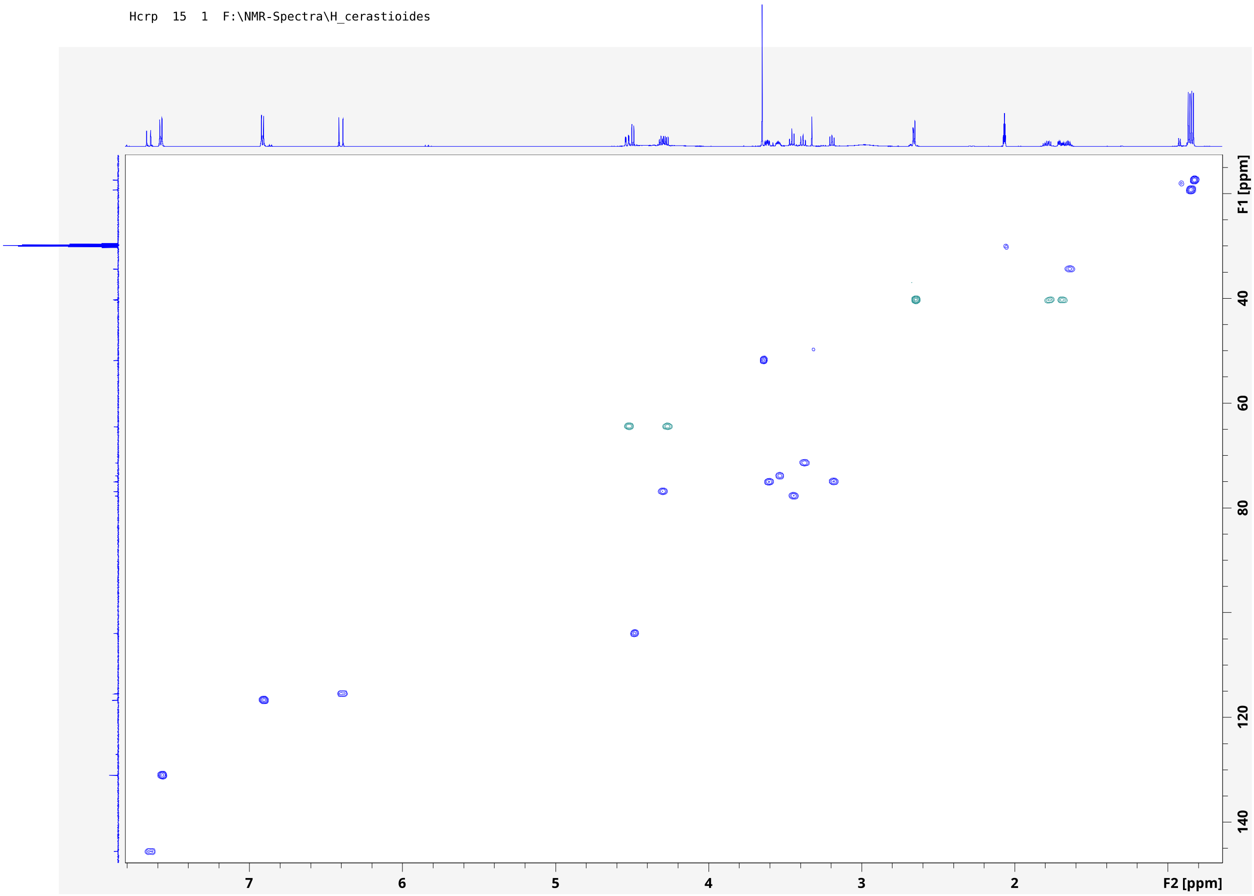

Figure S20. HSQC spectrum of compound **HC7**.

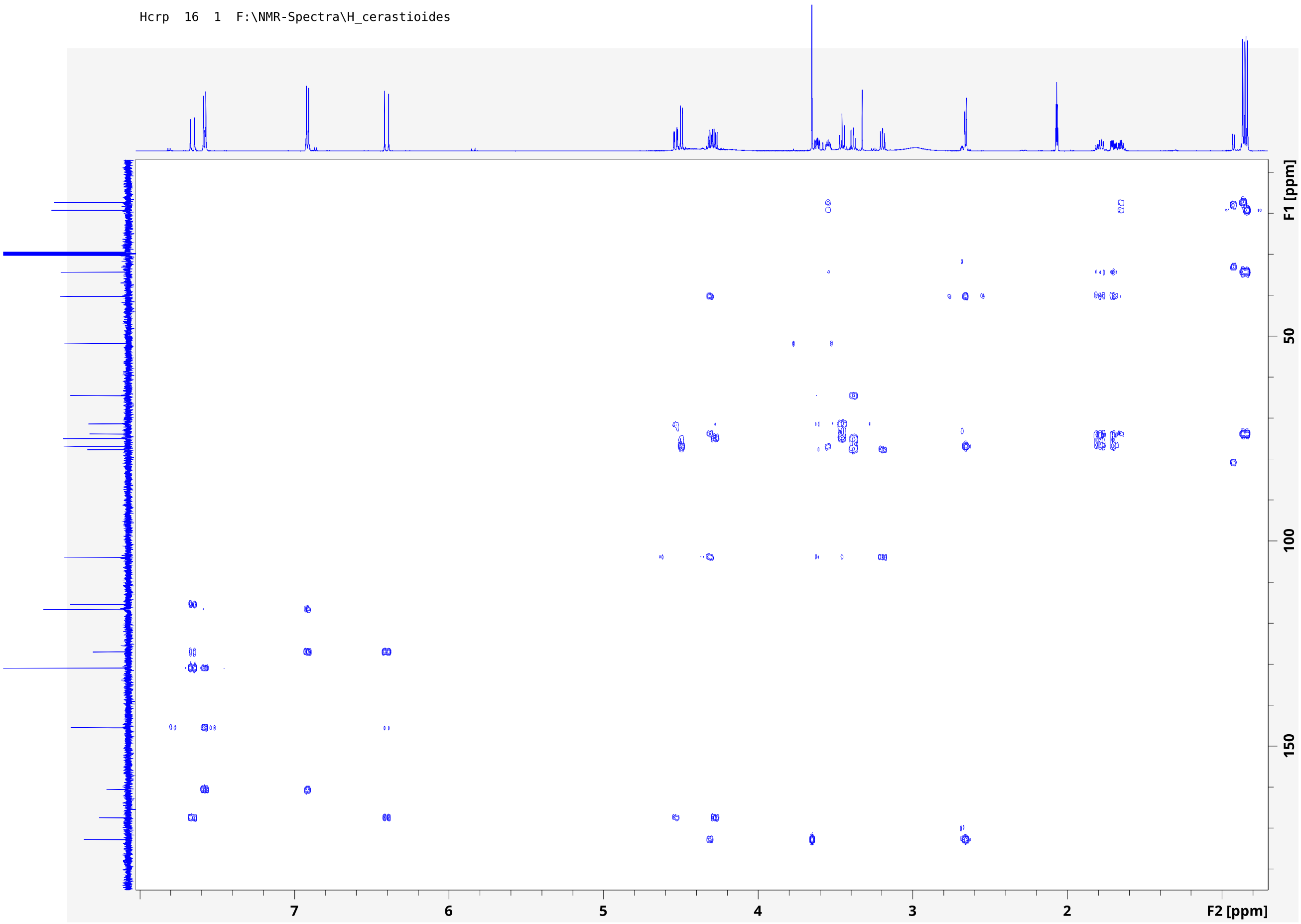

Figure S21. HMBC spectrum of compound **HC7**.
