# Supplementary material for: Phenolic Compounds from Hypericum cerastoides (Spach) N. Robson: Dereplication via UHPLC-HRMS/MS, Isolation, Identification, and Preliminary Biological Evaluation Focusing on Radical-Scavenging, Anti-α-Glucosidase, and Pro-Lipase Activities
Source: Metabolites. 2025 Sep 25;15(10):643. doi: 10.3390/metabo15100643 (PMC12565843; doi:10.3390/metabo15100643)
Supplement: Supplementary file 1 [file metabolites-15-00643-s001.zip › Figures_S22-S27.pdf]

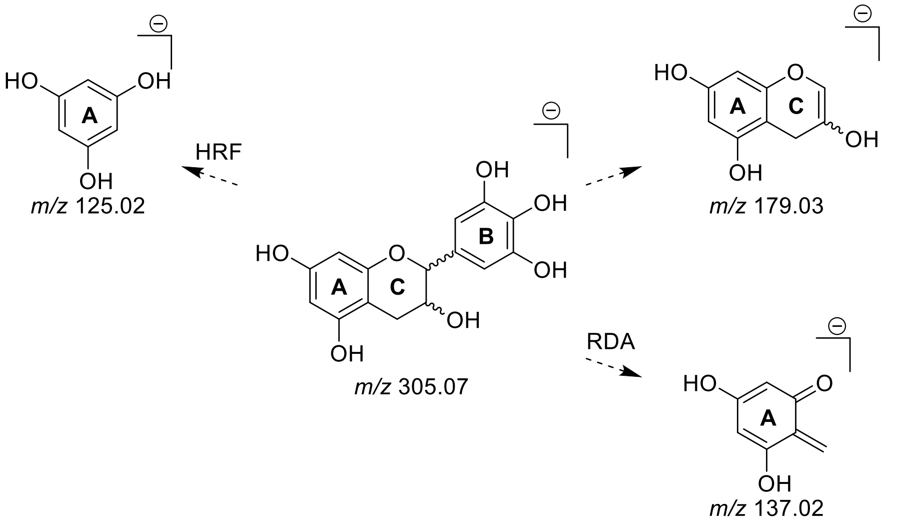

Figure S22. A plausible fragmentation pattern for compounds **1** and **2**.

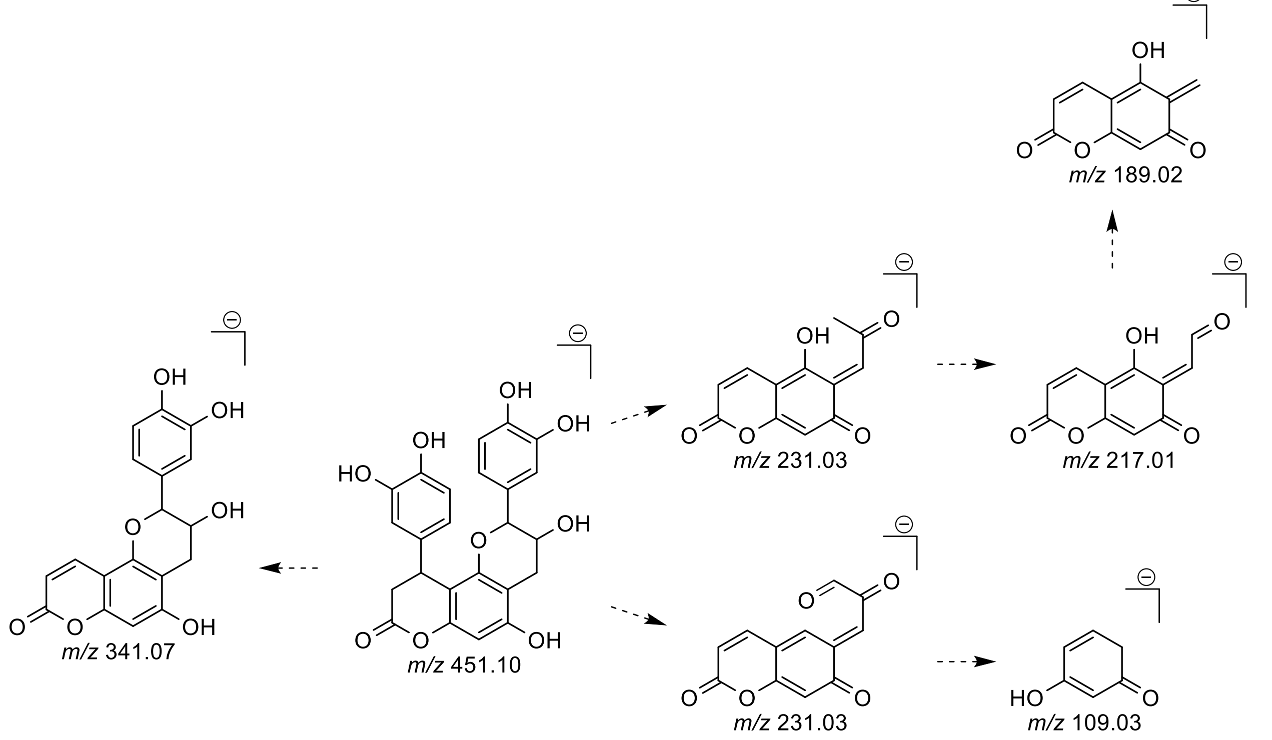

Figure S23. A plausible fragmentation pattern for compound **31**.

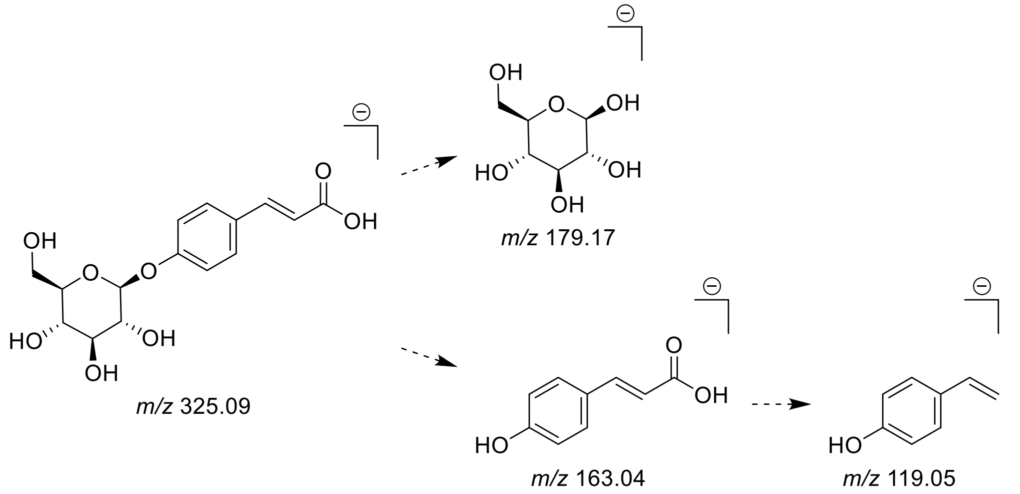

Figure S24. A plausible fragmentation pattern for compound **5**.

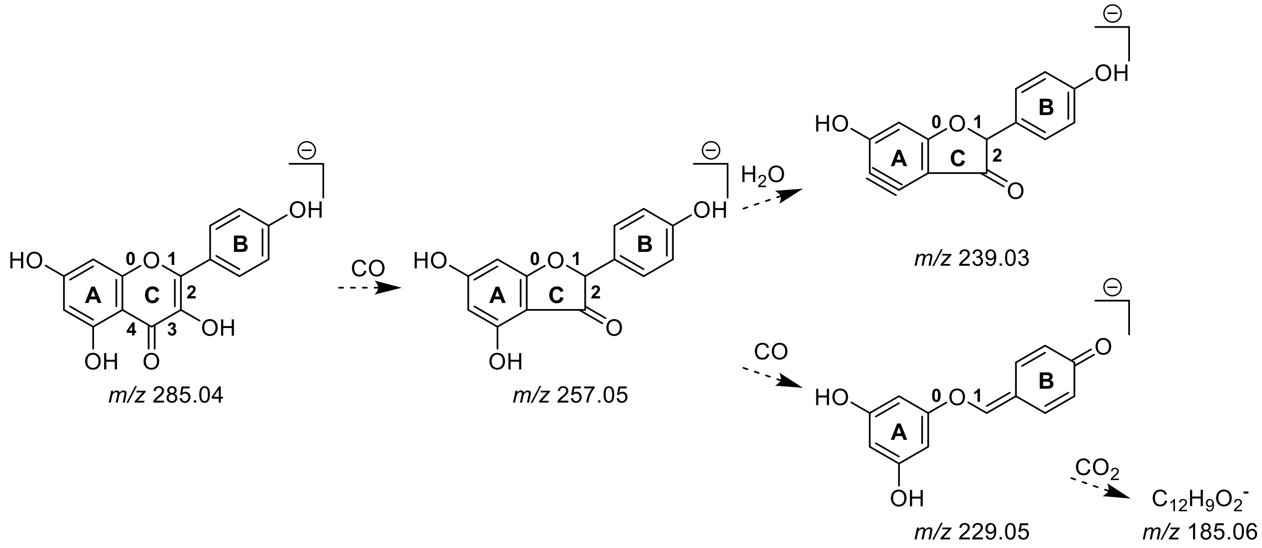

Figure S25. A plausible fragmentation pattern for compound **37**.

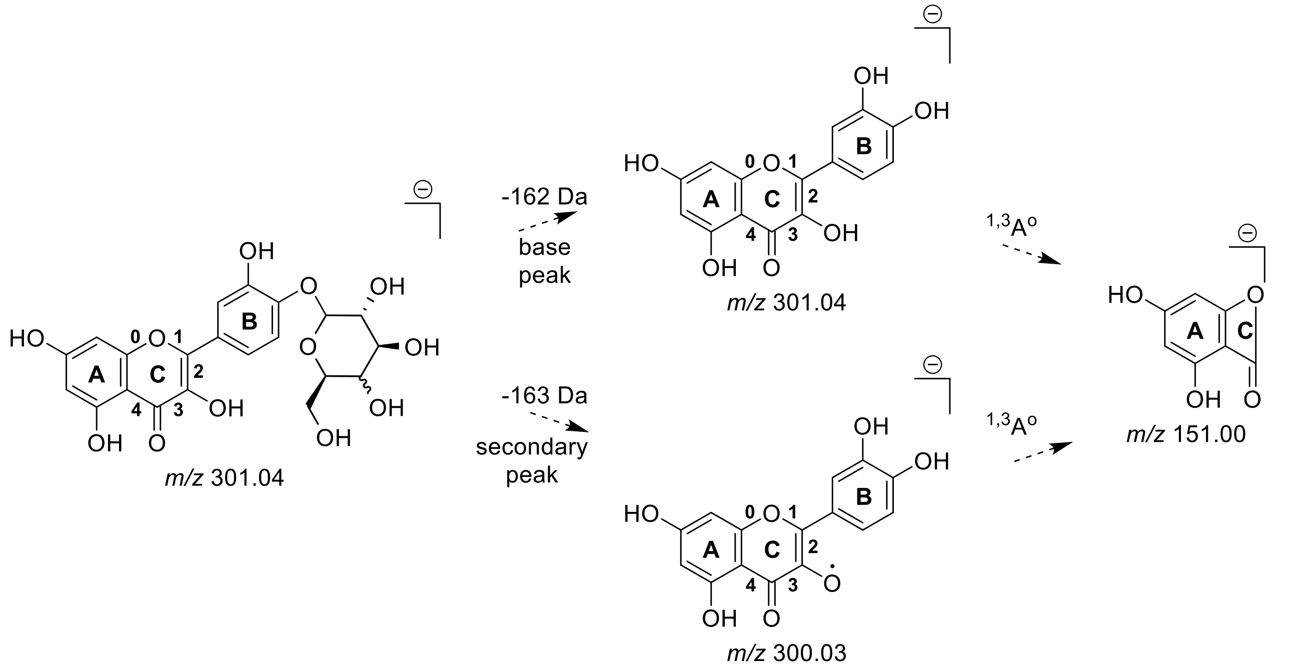

Figure S26. A plausible fragmentation pattern for compounds **29** and **34**.

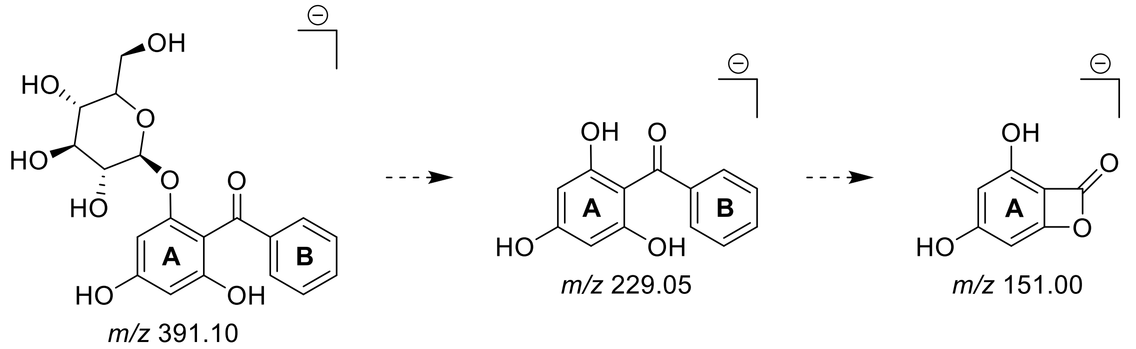

Figure S27. A plausible fragmentation pattern for compound **14**.
