# Supplementary material for: Phenolic Compounds from Hypericum cerastoides (Spach) N. Robson: Dereplication via UHPLC-HRMS/MS, Isolation, Identification, and Preliminary Biological Evaluation Focusing on Radical-Scavenging, Anti-α-Glucosidase, and Pro-Lipase Activities
Source: Metabolites. 2025 Sep 25;15(10):643. doi: 10.3390/metabo15100643 (PMC12565843; doi:10.3390/metabo15100643)
Supplement: Supplementary file 1 [file metabolites-15-00643-s001.zip › Table_S1.pdf]

Table S1. The detected and identified polar phenolic compounds, along with their quantities, in the EtOH extract from the aerial parts of *H. cerastoides*.

No	t_R (min)	Compound	Class[1]	Exact Mass	Δppm	Ion type	Molecular Formula	MS/MS product ions (intensity in %)	μg/g D.W. ±SD	Calc.[2]
1	2.87	gallocatechin	FLO	305.0669	4.41	$[M-H]^-$	$C_{15}H_{13}O_7$	125.02(100), 305.07(40), 179.03(28), 137.02(25), 109.03(10), 203.03(5), 287.05(1)	87.27±11.17	H
2	4.98	galloepicatechin	FLO	305.0670	4.51	$[M-H]^-$	$C_{15}H_{13}O_8$	125.02(100), 305.07(32), 179.03(25), 137.02(24), 109.03(9), 203.03(3)	34.22±0.53	H
3	5.72	catechin	FLO	289.0717	3.73	$[M-H]^-$	$C_{15}H_{13}O_6$	289.07(100), 245.08(65), 109.03(50), 125.02(42), 203.07(42), 151.04(22), 137.02(22), 179.03(21), 271.06(3)	193.22±1.12	H
4	5.81	5-*O*-*trans*-*p*-caffeoylquinic acid (chlorogenic acid)	HCA	353.0877	2.77	$[M-H]^-$	$C_{16}H_{17}O_9$	191.06(100), 192.06(4), 179.03(1), 135.04(0.3)	65.36±2.54	C
5	6.30	4-*O*-β-D-glucosyl-4-coumaric acid	HCA	325.0931	4.06	$[M-H]^-$	$C_{15}H_{17}O_8$	119.05(100), 163.04(12), 179.17(1)	25.47±0.16	C
6	7.30	procyanidin B2	FLO	577.1357	2.89	$[M-H]^-$	$C_{30}H_{25}O_{12}$	125.02(100), 289.07(79), 407.08(74), 161.02(26), 245.08(21), 137.02(15), 425.09(9)	132.02±2.72	H
7	8.18	5-*O*-*cis*-*p*-caffeoylquinic acid	HCA	353.0879	3.43	$[M-H]^-$	$C_{16}H_{17}O_9$	191.06(100), 192.06(4), 179.03(1), 135.04(0.5)	77.60±0.09	C
8	8.23	5-*O*-*trans*-*p*-coumaroylquinic acid	HCA	337.0932	4.26	$[M-H]^-$	$C_{16}H_{17}O_8$	191.06(100), , 163.04(8), 119.05(3)	39.63±0.19	C
9	8.38	Epicatechin	FLO	289.0719	4.39	$[M-H]^-$	$C_{15}H_{13}O_6$	289.07(100), 245.08(68), 109.03(55), 125.02(47), 203.07(39), 151.04(26), 137.02(23), 179.03(21), 271.06(4)	80.92±0.20	H
10	10.00	5-*O*-feruloylquinic acid	HCA	367.1037	3.65	$[M-H]^-$	$C_{17}H_{19}O_9$	191.06(100), 193.05(6), 134.04(4)	136.98±3.23	C
11	10.88	5-O-*cis*-*p*-coumaroylquinic acid	HCA	337.0933	4.38	$[M-H]^-$	$C_{16}H_{17}O_8$	191.06(100), 163.04(2), 119.05(1)	22.66±0.34	C

12	11.01	myricetin-3-*O*-galactoside	FLA	479.0828	1.59	$[M-H]^-$	$C_{21}H_{19}O_{13}$	316.02(100), 479.08 (28), 317.03(22), 271.03(11), 287.02(4), 178.99 (4)	259.44±0.09	H
13	11.18	myricetin-*O*-rutinoside	FLA	625.1406	1.11	$[M-H]^-$	$C_{27}H_{29}O_{17}$	316.02(100), 625.14(39), 317.03(21), 178.99 (6)	67.13±0.17	H
14	11.37	garcimanosone D	BEN	391.1033	2.33	$[M-H]^-$	$C_{19}H_{19}O_{9}$	229.05(100), 151.00(7.3)	83.93±0.99	M
15	11.39	myricetin-3-*O*-glucoside	FLA	479.0829	1.81	$[M-H]^-$	$C_{21}H_{19}O_{13}$	316.02(100), 479.08 (30), 317.03(22), 271.03(10), 287.02(5), 178.99 (4)	175.45±0.45	H
16	12.68	myricetin-*O*-(malonyl)-hexoside	FLA	565.0837	2.14	$[M-H]^-$	$C_{24}H_{21}O_{16}$	316.02(100), 521.09(29), 317.03(18), 178.99 (4)	157.26±2.87	H
17	13.77	quercetin-3-*O*-galactoside (hyperoside)	FLA	463.0886	3.17	$[M-H]^-$	$C_{21}H_{19}O_{12}$	300.03(100), 301.04(46), 463.09(29), 271.03(13), 255.03(7), 243.03(1), 151.00(6)	234.27±0.07	H
18	13.81	quercetin-3-*O*-rutinoside (rutin).	FLA	609.1457	1.06	$[M-H]^-$	$C_{27}H_{29}O_{16}$	300.03(100), 301.04(51), 609.15(47), 271.03(10), 255.03(6), 243.03 (1)	470.62±1.80	H
19	14.15	quercetin-3-*O*-glucoside (isoquercitrin)	FLA	463.0882	2.29	$[M-H]^-$	$C_{21}H_{19}O_{12}$	300.03(100), 301.04(53), 463.09(28), 271.03(13), 255.03(7), 243.03(1), 151.00(5)	470.81±1.34	H
20	14.30	myricetin-7-*O*-galactoside	FLA	479.0829	1.88	$[M-H]^-$	$C_{21}H_{19}O_{14}$	479.08 (100), 317.03(80), 316.02(55), 178.99 (24), 151.00(13)	34.90±0.38	H
21	14.50	myricetin-7-*O*-glucoside	FLA	479.0827	1.37	$[M-H]^-$	$C_{21}H_{19}O_{13}$	317.03(100), 479.08 (55), 316.02(24), 178.99 (21), 151.00(13)	27.99±0.54	H
22	14.86	quercetin-*O*-pentoside	FLA	433.0780	3.35	$[M-H]^-$	$C_{20}H_{17}O_{11}$	300.03(100), 301.03(26), 433.08(23), 271.03(11), 151.00(3)	99.57±2.39	H
23	14.94	quercetin-3-*O*-(6''-*O*-acetyl)-galactoside	FLA	505.0987	2.11	$[M-H]^-$	$C_{23}H_{21}O_{13}$	300.03(100), 301.03(44), 505.10(24), 271.03(15), 255.03(7), 243.03 (2), 463.09(3)	72.98±0.71	H
24	14.95	kaempferol-3-*O*-glucoside (astragalin)	FLA	447.0930	1.79	$[M-H]^-$	$C_{21}H_{19}O_{11}$	284.03(100), 447.09(69), 255.03(33), 285.04(30), 227.03(13)	18.57±0.07	H
25	14.96	trihydroxybenzophenone-*O*-(acetyl)-glucoside	BEN	433.1137	1.79	$[M-H]^-$	$C_{21}H_{21}O_{10}$	229.05(100), 151.00(6), 391.10(1)	143.89±0.91	M

		(acetyl-garcimangosone D)								
26	15.06	kaempferol-O-hexosylrhamside	FLA	593.1517	2.64	[M−H]⁻	$C_{27}H_{29}O_{15}$	285.04 (100), 284.03 (58), 257.05 (4), 229.05 (3), 239.05 (1), 416.60 (1)	27.94±0.03	H
27	15.30	quercetin-3-*O*-(2''-*O*-acetyl)-glucoside	FLA	505.0994	3.42	[M−H]⁻	$C_{23}H_{21}O_{13}$	300.03(100), 301.03(28), 505.10(22), 271.03(15), 255.03(7), 445.08(6), 243.03(1)	58.88±0.70	H
28	15.34	kaempferol-*O*-hexoside	FLA	447.0936	3.25	[M−H]⁻	$C_{21}H_{19}O_{11}$	284.03(100), 447.09(75), 285.04(46), 255.03(36), 227.03(15)	116.54±2.49	H
29	15.35	quercetin-4'-*O*-galactoside	FLA	463.0880	1.90	[M−H]⁻	$C_{21}H_{19}O_{12}$	301.04(100), 151.00(26), 178.99(18), 300.03(15), 463.09(11)	34.34±0.79	H
30	15.44	quercetin-*O*-rhamnoside (quercitrin)	FLA	447.0937	3.33	[M−H]⁻	$C_{21}H_{19}O_{11}$	300.03(100), 301.04(89), 447.09(31), 271.03(14), 151.00(9), 178.99(8), 255.03(8)	28.23±0.54	H
31	15.50	cinchonain Ib	FLI	451.1041	3.79	[M−H]⁻	$C_{24}H_{19}O_{9}$	341.07(100), 217.01(28), 189.02(19), 109.03(11), 231.03(10), 451.10(7)	112.36±2.14	H
32	15.73	myricetin-*O*-hexoside	FLA	479.0825	0.92	[M−H]⁻	$C_{21}H_{19}O_{13}$	317.03(100), 178.99 (17), 151.00(12), 479.08 (11), 316.02(10)	36.32±0.47	H
33	15.92	myricetin	FLA	317.03	2.68	[M−H]⁻	$C_{15}H_{9}O_{8}$	317.03(100), 151.00(56), 178.99(48), 137.02(35), 107.01(7), 193.01(3), 165.02 (3)	68.25±0.74	H
34	16.19	quercetin-4'-*O*-glucoside	FLA	463.0880	1.90	[M−H]⁻	$C_{21}H_{19}O_{12}$	301.04(100), 463.09(35), 151.00(23), 178.99(18), 300.03(14)	46.84±0.52	H
35	16.83	norathyriol	XAN	259.0249	4.73	[M−H]⁻	$C_{13}H_{7}O_{6}$	259.03(100), 215.03(3), 187.04(2)	66.65±0.77	M
36	17.87	quercetin	FLA	301.0353	3.42	[M−H]⁻	$C_{15}H_{9}O_{7}$	301.04(100), 151.00(92), 178.99(41), 121.03(17), 107.01(13), 193.01 (2), 149.02 (2)	175.08±2.20	H

37	19.47	kaempferol	FLA	285.0403	3.44	$[M-H]^-$	$C_{15}H_9O_6$	285.04(100), 257.05 (1), 239.03 (1), 229.05 (1), 185.06 (1)	52.83±0.69	H
38	20.00	3,8'-biapigenin	FLD	537.0829	2.46	$[M-H]^-$	$C_{30}H_{17}O_{10}$	151.00(100), 385.07(49), 443.04(23), 417.06(4)	157.12±1.41	H
39	20.71	3',8''-biapigenin (amentoflavone)	FLD	537.0826	1.78	$[M-H]^-$	$C_{30}H_{17}O_{10}$	375.05(100), 537.08(68), 417.06(28), 443.04(13)	45.86±0.74	H

[1] Classes of secondary metabolites: BEN – Benzophenones; HCA – Hydroxycinnamic acids; FLO – Flavan-3-ols (catechins); XAN – Xanthones; FLI – Flavonolignans; FLA – Flavonols; FLD – Flavone dimers. [2] The quantity of the metabolites was calculated as: C – chlorogenic acid, M – mangiferin, and H – hyperoside.
